# Improving health, wellbeing and parenting skills in parents of children with special health care needs and medical complexity – a scoping review

**DOI:** 10.1186/s12887-019-1648-7

**Published:** 2019-08-30

**Authors:** Sally Bradshaw, Danai Bem, Karen Shaw, Beck Taylor, Christopher Chiswell, Mary Salama, Eve Bassett, Geetinder Kaur, Carole Cummins

**Affiliations:** 1 Institute of Applied Health Research, College of Medical and Dental Sciences, Murray Learning Centre, University of Birmingham, Edgbaston, Birmingham, B15 2TT UK; 2 Birmingham Women and Children’s NHS Foundation Trust, Steelhouse Lane, Birmingham, B4 6NH UK

**Keywords:** Children with special health care need, Children with medical complexity, Routine health care contact, Parents, Health, Wellbeing, Intervention, Scoping

## Abstract

**Background:**

Parenting children with special health care needs can be challenging particularly if children have complex conditions. Parents may struggle to manage their child’s health and their own emotions, contributing to poorer health outcomes for the family. Frequent healthcare contact presents opportunities to intervene, but current evidence review is limited. This review scopes and synthesizes interventions to improve health, wellbeing and parenting skills.

**Methods:**

Using formal scoping review methodology MEDLINE, EMBASE, PsycINFO, CINAHL, The Cochrane Library, ERIC, ASSIA, HMIC and OpenGrey were searched to February 2017. Citations were double screened according to predetermined eligibility criteria. Data were extracted and synthesized on study design, population, measurement tools, and results.

**Results:**

Sixty-five studies from 10,154 citations were included spanning parenting programs, other parent behavior change interventions, peer support, support for hospital admission and discharge and others. Interventions for parents of children with a wide range of conditions were included. These targeted a broad selection of parent outcomes, delivered by a wide variety of professionals and lay workers. Most studies reported positive outcomes. No serious adverse events were noted but issues identified included group and peer relationship dynamics, timing of interventions in relation to the child’s disease trajectory, the possibility of expectations not fulfilled, and parent’s support needs following intervention. Children with medical complexity were not identified explicitly in any studies.

**Conclusions:**

The range of interventions identified in this review confirms that parents have significant and diverse support needs, and are likely to benefit from a number of interventions targeting specific issues and outcomes across their child’s condition trajectory. There is much scope for these to be provided within existing multi-disciplinary teams during routine health care contacts. Careful tailoring is needed to ensure interventions are both feasible for delivery within routine care settings and relevant and accessible for parents of children across the complexity spectrum. Further review of the existing literature is needed to quantify the benefits for parents and assess the quality of the evidence. Further development of interventions to address issues that are relevant and meaningful to parents is needed to maximize intervention effectiveness in this context.

**Electronic supplementary material:**

The online version of this article (10.1186/s12887-019-1648-7) contains supplementary material, which is available to authorized users.

## Background

Children with special healthcare needs (CSHCN) are a growing group of children who have or are at increased risk of chronic physical, developmental, behavioral or emotional conditions who require healthcare and related services of a type or amount beyond that required by children generally [1]. This definition is broad and can range from children with a single condition to children with medical complexity (CMC) who are characterized as children who have substantial family-identified service needs, multiple chronic and severe conditions, functional limitations, and high health care use [2]. This can result in frequent emergency department use, long hospitalizations, and frequent readmissions [3].

Three stages of parental adjustment to their children’s chronic disease have been described: crisis (pre-diagnosis and initial adjustment), chronic (the long haul), and terminal phases in progressive conditions [4] often accompanied by increased healthcare use. While many parents will adjust to their child’s conditions [5, 6], others will experience ongoing distress that can be associated with poorer health outcomes for parents, the child and other family members [7]. Increased healthcare use offers opportunities to identify and respond to parental support needs. Providing effective interventions within routine care settings may be a particularly feasible and effective way of offering support when parents have increased support needs.

A broad range of interventions have been designed to address parent and caregiver health, wellbeing and parenting from a range of disciplinary and theoretical perspectives. Relevant reviews of evidence have often addressed narrow intervention approaches, for example psychological interventions [8] and interactive media [9]. Other potentially important intervention approaches, for example addressing social support, and support with common parenting issues have been less well reviewed. Given this, we carried out a scoping review to identify interventions, where parent outcomes have been measured, targeted at improving outcomes for parents of CSHCNs, including parents of CMC.

This scoping review identified, characterized and synthesized interventions for parents and families of CSHCN, limited to children with chronic physical conditions to ensure a manageable scope. The objectives were to describe the interventions, study populations, intervention targets and measures, reported efficacy or comparative effectiveness; and to explore the extent results are relevant and transferable to delivery within routine health care settings for both parents of CSHCN and, within that, of CMC.

## Methods

### Review methodology

We used a scoping review methodology, drawing on Arksey and O’Malley’s methodological framework [10], informed by Joanna Briggs Institute Guidance [11], because it is ideal for identifying and summarizing a broad range of studies that have used diverse research methodologies. The six scoping review stages of were followed; framing the research question, identifying relevant studies, study selection, charting the data, collating and summarizing, and reporting the results. A full protocol has been published [12]. There were three minor variations to the published protocol: the research question was reworded slightly to add clarity following initial exploratory searches; non-English language studies were included; reference list screening was not undertaken due to the unexpectedly large number of eligible studies identified through primary searches.

### Search strategy

Exploratory searches were first performed on MEDLINE and CINAHL using a combination of relevant text words and index terms. There was extensive piloting of search strategies drawing on existing literature. Initial search results were screened for additional key words and the search strategy was optimized for each database, using database-specific subject headings. Following this exercise MEDLINE, EMBASE, PsycINFO, CINAHL, The Cochrane Library, ERIC, and ASSIA were searched with no language restrictions from inception to February 2017. HMIC and OpenGrey were searched for grey literature. Key search terms are shown in Table 1 and the full MEDLINE search, which was adapted to each database, can be found in Additional file 1.

**Table 1:** Key search terms

Key concept	Key words
Parents	carer, family, father, mother, parent
Child	adolescent, babies, baby, child, children, juvenile, kid, minor, neonate, pediatric, teen, toddler, youth
Chronic childhood disease	activity limiting, Children with special health care needs, chronic, condition, disease, disorder, illness, long term, medical complexity, pain, sickness
Outcomes	adaptation, psychological, coping, emotional adjustment, health, quality of life, self-concept, self-efficacy, social adjustment, stress, resilience, wellbeing,
Intervention	education, intervention, program, skills, training

The search results were imported to EndNote X7, and the duplicates were removed. Titles and abstracts were independently screened by two researchers, with discrepancies reviewed by a third. Full texts were retrieved for all potentially relevant abstracts and assessed against the inclusion criteria by the primary author, and double checked by a second author. Eligible protocols were reviewed periodically and eligible full texts were included when published.

### Inclusion criteria

Interventions for parents of CSHCN, with any or no comparator, aimed at improving wellbeing and parenting skills were eligible. Any study designs where parents were directly involved in the intervention, and where outcomes were assessed and measured in parents, were included. “Parents” were defined as anyone with parenting or caring responsibility for CSHCN up to the age of 18, who could be in hospital or at home. Studies could be condition specific or non-condition specific and undertaken in any setting (e.g. acute, primary care, community) if the intervention was adaptable to delivery by generalist healthcare staff and / or lay workers, within routine care settings, by existing health care teams. Adaptability was judged by whether general staff and / or lay workers could reasonably be trained to deliver the intervention. For example manualized psychological interventions with a description of standardized training for general and / or lay staff were included, but psychologist or psychiatrist delivered psychotherapy interventions were not.

### Exclusion criteria

Studies were excluded if they did not report parent outcomes or they were not adaptable to delivery by generalist healthcare staff or lay workers (for example specialist psychotherapy techniques). We also excluded interventions focused primarily on acute conditions or end of life care, parents with long-term conditions, or children with behavioral, emotional or mental health conditions (e.g. Attention Deficit Hyperactivity Disorder (ADHD), autism, depression) without physical co-morbidities.

### Data extraction

Data were extracted by the first author and double checked by an additional member of the study team. Multiple publications were found for a number of interventions. Where this was the case, the paper that reported parent outcomes was used primarily, with information from additional papers used where necessary, and indicated in the reference list. Most data were grouped into categories to allow key characteristics to be described across the papers, but where data reflected wider relevant issues these were labelled and grouped using the principles of directed qualitative content analysis [13] in order to identify patterns consistent with relevant literature. These included the child’s condition including complexity based on Cohen’s definition [2], intervention descriptions, outcome measures, and outcomes.

Intervention descriptions were assessed according to the Template for Intervention Description and Replication (TiDieR) framework which outlines the reporting domains necessary for interventions to be described in sufficient detail to allow their replication. Domains include what, why, who, how, where, when and how much, modifications, tailoring and fidelity [14]. Outcome measures were labelled as single (e.g. anxiety) or multiple domain outcomes (e.g. depression and anxiety), before grouping by construct. Study results were summarized as fully positive, mixed (if some results favored the intervention but not all), or fully negative. Evidence of harm was coded and grouped into common themes.

## Results

**Fig. 1:**
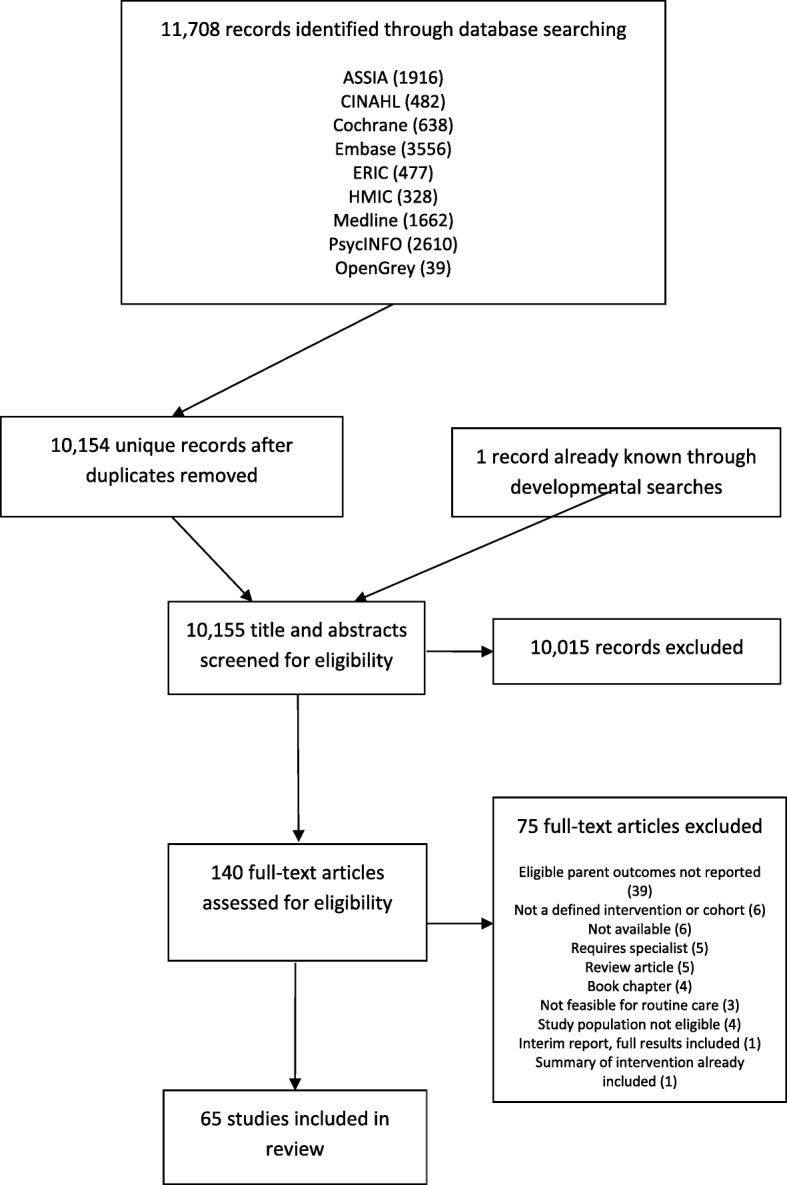
PRISMA Chart

The literature searches identified 10,155 unique records of which 140 full texts were assessed for eligibility following title and abstract screening. Sixty-five studies were included in the review. The study selection process is illustrated by the Preferred Reporting Items for Systematic Reviews and Meta-Analyses (PRISMA) flow diagram in Fig. 1. Additional file 2 contains a list of studies excluded at the full text screening stage with reasons. A summary of data extracted for each study can be found in Additional file 3.

### Study design

Study design characteristics are summarized in Table 2. The majority of studies were quantitative (*n* = 55) using trial (RCT) (*n* = 42, 64%) or observational designs. (*n* = 13, 23%) In general sample sizes were small to medium, and this review includes data from a total of 3856 parents or family units. The median sample size was 60, ranging from 6 to 380. Follow-up periods were often short term, and seldom measured outcomes past 6 months.

**Table 2:** Study design characteristics

Study design (n)	Sample size and (n)	Follow up period
RCT (23) Quantitative observational (13) Mixed Methods Evaluation (4) Qualitative evaluation (5) RCT pilot (6) RCT abstract (4) RCT protocol (4) RCT secondary analysis (2) Non-randomized controlled trial (2) Cluster RCT (1)	< 10 (3) 10–50 (24) 50–100 (16) 100–200 (18) > 200 (2) Not stated (1)	< 1 month (7) 1–3 months (15) 3–6 months (6) 4–6 months (20) 7–12 months (6) > 13 months (2) Unclear (7) Varies (1)

### Other study characteristics

Table 3 summarizes key population and intervention characteristics across the studies. Three quarters (*n* = 49, 75%) were open to any parent or caregiver but a notable proportion were open to mothers and female caregivers only (*n* = 6, 9%) whilst none targeted only fathers. Despite parental support needs changing as children grow and develop half of the studies (*n* = 32, 49%) did not specify target or include children’s ages, and appeared to be open to parents of children of any age. Interventions were most commonly delivered to parents of children with established long term health needs in the community or outpatient settings (*n* = 41, 63%) or during hospital admission (*n* = 17, 26%). Approximately a third (*n* = 24, 37%) of interventions did not specify eligible children’s health conditions and, of those targeting single conditions, diabetes (*n* = 6), cancer (*n* = 4) and asthma (*n* = 3) were most common.

**Table 3:** Key study characteristics

Target population (n)	Child diagnosis (n)		Country (n)	Intensity and format	Agent
Eligible parents Any parents or caregivers (49) Mothers (9) Families (5) Mother and female relatives (1) Parents and grandmothers (1)	Not specified (24)	Rare diseases (2) Cancer, asthma and diabetes (1) Chronic kidney disease (1) Coronary heart disease (1) Cystic fibrosis (1) Eczema (1) Epilepsy (1) Hematology and oncology (1) Hemophilia (1) Rett Syndrome (1) Sickle cell disease (1) Single ventricular defects (1) Technology assisted lung disease (1) Thalassemia (1)	US (24) UK (9) Australia (6) Canada (6) Germany (3) Switzerland (3) Iceland (2) Sweden (2) Chile (1) China (1) Denmark (1) Iran (1) Japan (1) Malaysia (1) Mexico (1) New Zealand (1) Spain (1) Thailand (1)	Intensity < 1 h (4) 1–2 h (3) 3–5 h (6) 6–10 h (16) 11–16 h (7) 17–24 h (6) > 24 h (2) Unclear (8) Varies (13)	Unclear (14) Multi-disciplinary team (11) Peers (7) Nurse (6) Written / audio / visual (6) Psychologist (4) Health professional and peers (3) Counsellor (2) Other – qualified (2) Program specific trained practitioner (2) Psychologist and peers (2) Doctor (1) Financial counsellor (1) Massage therapist (1) Psychiatrist (1) Qualified non-specialist (1) Doctor and charity (1)
	Multiple specified conditions Asthma and / or allergies and / or eczema (1) Autism Spectrum Condition and intellectual and/or complex disabilities (1) Cystic fibrosis, spina bifida or diabetes (1) Life-threatening conditions (1) Lupus erythematosus and nephritis (1) Type 1 Diabetes and Irritable Bowel Disease (1)				
Age of child Child age specified (33) Child age not specified (32)					
Care trajectory point Long term community / outpatients (41) During hospital admission (17) Before elective admissions (2) Both inpatients and outpatients (2) Hospital admission (1) New diagnosis (1) Not clear (1)	Single conditions Type 1 Diabetes (6) Cancer (4) Asthma (3) Chronic pain (2) Disabilities (2) Juvenile Rheumatoid Arthritis (2)			Format Face-to-face (47) Remote (12) Both (4) Not clear (1) Online (5) Telephone (4) Recorded information (2) Email (1) Mixed (2) Videoconferencing (1) Workbook (1) Interactive (60) Not interactive (4) Not clear (1)	

When assessed against Cohen’s four dimensions of medical complexity [2], only one study was likely to include a significant proportion of CMC, an intervention for parents of children with chronic lung disease requiring technological assistance [15]. Inclusion criteria suggest that parents of CMC would have been eligible to participate in an additional 20 (31%) interventions [15–34] but condition severity and complexity were not discussed within these studies. No information was available on whether and how parents of more complex children were included, and whether intervention suitability and outcomes varied by the complexity of the child’s condition.

Interventions were most commonly from the US (*n* = 24), followed by the UK (*n* = 9), Australia (*n* = 6) and Canada (*n* = 6). Five studies (8%) were from middle income countries, China [35], Iran [36], Malaysia [28], Mexico [37], and Thailand [38], and there were no examples from low income countries. Many (*n* = 29, 45%) interventions were delivered in 10 h or less, and only two interventions (3%) were delivered over more than 24 h. Most interventions included some face-to-face contact (*n* = 59, 90%) with opportunities for person-to-person interaction in 60 interventions (92%).There were a wide range of intervention agents; more than one member of a multi-disciplinary team (*n* = 11, 17%), peer (*n* = 7, 11%), nurse (*n* = 6, 9%) and pre-written written or recorded audio or visual materials (*n* = 6, 9%).

### Intervention scope

Included studies can be grouped into five main categories: parenting programs; other targeted parent behavior change interventions; peer support; preparation and support for hospital admission or discharge; and other interventions.

#### Parenting programs

There were five parenting programs for parents of children with different chronic diseases, including bespoke programs [23, 39], and Triple-P based parenting programs [40–42]. These are all based on the premise that parenting programs can reduce the risk of child behavioral, emotional or psychological problems, and improve both child and parent outcomes.

#### Targeted parent behavior change

Twenty-seven interventions aimed to change aspects of parenting behavior including: Acceptance and Commitment Therapy (ACT) [43, 44], Cognitive Behavioral Therapy (CBT) [37, 45], Problem Solving Skills Therapy (PSST) [46, 47], coping skills training [48], multi-family therapy [24], individual family therapy [49], adapted Chronic Disease Self-Management based programs [25, 26], group meetings [27–29, 38, 50–54], residential programs [22, 55–57], an interactive online application [58], participatory training [59], and filial therapy, based on play therapy [30, 60].

#### Peer support

In five interventions, the primary mechanism of action was the development of peer support, including online peer support groups [15, 61], an email list serve [62], telephone peer support groups [63], and one to one peer matching [16, 64].

#### Preparation and support for hospital admission or discharge

Twenty-six interventions aimed to provide support to parents around their child’s hospital admission; including pre-admission preparation [31, 32], information on admission [65–67], and post-discharge support [34, 68]. During hospital admission there were examples of psychosocial risk assessment [69], education [35], early palliative care (using palliative care principles but not provided as end-of-life care) [33, 70, 71], and structured exercise sessions [72, 73].

#### Other interventions

Other interventions included mother to child massage training [36, 74], mindfulness / relaxation [18, 19], narrative therapy [20, 75, 76], therapeutic conversations [77, 78], wish granting [21], communication skills training [79], and financial counselling [80].

### Intervention targets and outcome measures

There were a broad range of intervention targets. These included increasing adaptation and adjustment, competence, confidence, coping, empowerment, health, positive functioning, positive emotions, self-efficacy, self-care, self-regulation, self-satisfaction, psychological flexibility, psychological health, quality of life, resilience, and wellbeing. Interventions also aimed to reduce anxiety, depression, stress, burnout, distress, negative emotions, performance-based self-esteem and post-traumatic stress disorder. Targets around the relationship between the parent, their child, wider support network or professionals were also mentioned including bonding, child behavior management, clinician-family communication, conflict within families or with health care teams, family functioning, parental acceptance of child, parent-child relationship, parenting practices and social support. Other targets included decision making, financial management, illness management, disease knowledge, problem solving skills, blood pressure and pulse rate and parent-child shared management.

Targets were operationalized in terms of outcomes and measures with little consistency. The most common outcome measured was anxiety, in 19 studies (29%). There were a total of 129 outcomes measured using 161 different tools. Outcomes measured five times or more are shown in Table 4 with tools used. Outcomes were most frequently assessed using validated measures of mental health, family functioning and quality of life. Less frequently measured were determinants of mental health and functioning. Determinants measured more than once included psychological flexibility, wellbeing, adjustment, family conflict, perceived social support and problem solving skills. The amount and breadth of outcomes was, however, much wider than this: 99 additional outcomes were measured only once. There was little evidence of consensus across the studies of what might constitute core outcomes for this group of parents. As expected and appropriate due to the nature of the outcomes, they were almost exclusively measured using parent self-reported outcome measures. The only exceptions to these were blood pressure [73], and researcher observations [22, 53, 65–67].

**Table 4:** Outcomes and measurement tools

Outcome (n, %)	Tools used (n)
Anxiety (20, 31)	State Trait Anxiety Inventory (9); State Trait Anxiety Inventory -state scale only (3); Generalized Anxiety Disorder 7 (2); Psychiatric Symptom Index (2); Beck Anxiety Inventory (1); State Anxiety Inventory (1); Taylor’s Manifest Anxiety Scale (1)
Stress (12, 18)	Acute Stress Disorder Scale (1); Pediatric Inventory for Parents (measures parenting stress) (1); Parental Stress Scale (1); Parental Stressor Scale: PICU (1); Parenting Stress Index (1); Parenting Stress Index-Short Form (2); Pediatric Stressor Scale: Pediatric Intensive Care (2); Perceived Stress Scales (1); SPSQ, Swedish version of PSI (1); Symptoms of stress inventory (1)
Depression (11, 17)	Beck Depression Inventory-II (3); Beck Depression Inventory (2); Center for Epidemiologic Studies Depression Scale (1); Center for Epidemiologic Studies Short Depression Scale (1); Depression (1); Depressiveness Scale, Complaints List (1); Patient Health Questionnaire 9 (1)
Coping (10, 15)	Coping Health Inventory for Parents (3); Family Crisis Oriented Personal Evaluation Scale (2); Brief Cope Inventory (1); Coping question (1); Issues in Coping With IDDM-Parent Scale (1); Researcher developed questions (1)
Family functioning (9, 14)	Family Adaption and Cohesion Evaluation Scale (1); Family Management Measure (1); Feetham Family Functioning Survey (2); Iceland Expressive Family Functioning Questionnaire (2); Researcher developed questions (1); Relationships between family members through family arches (1); McMasters Family Assessment Device (1)
Quality of Life (6, 9)	PedsQL Family Impact Module (2); 36-Item Short Form Survey (1); Pediatric Asthma Caregiver’s Quality of Life (1); Parents Diabetes Quality of Life Questionnaire (1); Quality of life (family impact scale) (1); World Health Organization Quality of Life – BREF (1)
Mood (6, 9)	Profile of Mood States (4); Brief Mood Rating Scale (1); Profile of Mood States Short Form (1)
Post-traumatic stress (5, 8)	Post Hospitalization Stress Index for Parents (2); Impact of Event Scale (1); Posttraumatic Stress Disorder Checklist – Specific (1); PTSD Checklist (1)

### Intervention descriptions

There was also variability in the extent and quality of intervention descriptions when assessed against the TiDieR Framework [14]. Almost all studies reported the rationale for their approach and included some information about what the intervention included. Other aspects were less well described. A large majority of studies (around 85%) described to some extent how, when and how much of an intervention was delivered, and around two thirds described who provided the intervention and where. Intervention fidelity and tailoring were less commonly addressed, in a quarter and a fifth of studies respectively. Less than 10% of all studies made any mention of whether or not modifications were made to intervention protocols during the study periods.

The vast majority of interventions targeted parents directly, and assumed a direct relationship between the intervention and outcomes measured. There were two exceptions where parents were central to the intervention but the mechanism of action depended indirectly on changes in professional behavior and referral to unspecified support that were assumed to be responsible for changing parent outcomes [69, 70]. These were included as parents were directly involved, and their outcomes were directly measured.

### Parent participation in research

Only eight studies (12%) mentioned involvement of parents in the intervention design process, and all but one [80] were published after 2011. Of these, four descriptions [38, 58, 59, 61] suggest that involvement of parents in the design process might have had a significant impact on the intervention. These studies describe the use of qualitative research methods to elicit parents’ needs and preferences in the design stages. In three more studies [25, 34, 80], the authors state that there was parent involvement though, from the descriptions available, the impact on intervention design is unclear. In one further study parent feedback led to two minor modifications in written information [42]. There was no evidence of parent participation in the selection of intervention targets and tools. One study did encourage parents to choose their own goals, and progress towards these was assessed as an aggregate outcome measure [23], but no studies provided any evidence of consultation with parents and families about what might be important and appropriate universal outcomes.

### Evidence of effectiveness

Half of the studies reported findings that were fully in favor of the intervention, (*n* = 31, 52%) with the rest producing mixed results. (*n* = 25, 42%) The exception for two RCTs where intervention parents had worse outcomes than control parents for all outcomes measured [70, 75]. Theory based interventions were associated with positive results, and the type of theory also appears to be important. Interventions that took a behavioral or cognitive based approach tended to report more favorable outcomes than those taking a family and ecological systems based approach. The few behavioral and cognitive based studies that did not report fully positive results (4/21, 19%) included two pilots which were not powered to detect significant changes [50, 59], and two adapted Triple P programs which did report improvements in parenting [40] and diabetes related outcomes [42]. In contrast 5 out of 6 family and ecological systems theory-based approaches (83%) reported mixed results [15, 16, 56, 63, 64], whilst almost half of the studies (6/13, 46%) that took a mixed approach encompassing behavioral cognitive and family and ecological systems theories reported fully positive findings.

### Evidence of harm

Although not routinely collected across the studies there were no reports of serious harm to participants. There were, however, several common themes that are important considerations for future interventions. There were issues related to group or peer dynamics. In one group tensions arose between parents of children with mainly physical disabilities and parents of children who had behavioral or emotional disorders [26], and in another study parents commented on a lack of similarity between their specific situation and that of the other parents [63]. Some participants struggled with incompatible peer relationships [15]. Sometimes interventions were not well matched to the parent’s prior experience: some parents felt an intervention to be ‘too late’ [50], while others felt that they had already dealt with the issues that were discussed [63], that group discussions were repetitive [50, 62], or that attendance had not resulted in new learning [26]. Exposure to an intervention too early may however raise issues that parents are not ready to face [49, 53].

Interventions sometimes did not fulfil expectations. Disappointments about the size and composition of support groups [45], poor attendance by other parents [26], and a lack of meaningful peer connection [15] were reported. There was also disappointment about the amount of information available [63], and perceived misinformation [62]. Some interventions raised expectations outside of their control that were associated with subsequent deterioration in perceived social support [78], and lower satisfaction with clinical care [70]. Other pragmatic issues were highlighted including high rates of parents unable to commit to interventions [25, 50], high drop-out rates and nonparticipation once enrolled [72], and feeling overwhelmed once enrolled in an intervention [62].

Some parents reported negative feelings after being exposed to others’ problems [76], or seeing other children meet milestones that their own child was unlikely to meet [15]. Sometimes the short term nature of interventions was seen as a risk due to the potential dangers of challenging existing coping strategies without providing long term support [20]. Parents in one study expressed concerns about being left alone and “cut off” after the intervention, feeling that they might lose the positive changes gained during the period the group met regularly [26].

## Discussion

This scoping review found a wide range of interventions for parents of CSHCN targeting multiple and diverse parent outcomes. The diversity of interventions and outcomes suggests that one approach is unlikely to meet the needs of all parents, and parents are likely to benefit from a number of interventions targeting specific issues and outcomes at different times in their child’s chronic disease trajectory. We aimed to assemble evidence of reported efficacy or comparative effectiveness; interventions were mostly evaluated using quantitative methods, with a sizeable proportion of RCTs suggesting that targeted systematic reviews assessing effectiveness might yield useful results. Outcome measures were heterogeneous, with 129 outcomes measured using 161 different tools, though the most common outcomes were anxiety, stress, depression and coping. While this makes summary synthesis difficult, almost all studies reported fully positive or mixed results. Caution is needed however, as studies generally included small sample sizes and short follow-up periods and a scoping review design does not incorporate full quality assessment.

No serious adverse events were noted but issues identified included group and peer relationship dynamics, timing of interventions in relation to the child’s disease trajectory, the possibility of expectations not fulfilled, and parent’s support needs following intervention. These are important issues to consider when attempting to create or adapt interventions for delivery alongside routine health care, where the ability to provide interventions for parents with similar specific situations, and the feasibility of providing support post-intervention might be limited. This is particularly relevant to parents of children towards the complex end of the CSHCN spectrum and who may face a combination of issues including managing their child’s health, disruption to family life, and reactions to their child’s diagnosis including feelings of loss, guilt, fear and shame [81]. Parents often provide constant highly specialized and intensive medical care that may result in sleep deprivation, social isolation, chronic distress and other mental health challenges, which may result in physical stress responses, withdrawal from paid work, higher risk of poverty and an intensifying cycle of caregiver stress [82]. Interventions will need careful tailoring to their’ circumstances to facilitate participation and satisfaction.

We sought to explore the extent to which results are relevant and transferable to delivery within routine health care settings. Variability in the quality of intervention descriptions, with missing information, means that although many studies reported positive results, both the fidelity of intervention implementation and transferability to other settings are uncertain. Where specified, interventions mostly targeted single or restricted conditions, with condition complexity or severity rarely described. The extent of under-reporting of interventions and populations is such that most would require substantial local tailoring, development and piloting before adoption.

Given the importance of tailoring to the needs of parents in routine care settings it is notable that parents were rarely involved in intervention development, and this needs to be addressed in future research to ensure that interventions are acceptable and the outcomes addressed are those that are important and meaningful to parents. There is much potential for research and clinical teams willing to take on that development work. It is disappointing that few studies included parental participation in research, as this is increasingly required for service development and research funding. That many interventions were delivered using standardized manual based courses, or written, audio or web-based information, and facilitated by many different professionals and lay workers, indicates that many could be delivered by members of existing multi-disciplinary teams. Examples of remote and flexible interventions, in addition, demonstrate that geography or busy schedules do not need to be a barrier to delivery or parent participation.

This review is based on a broad and thorough literature search resulting in a large body of evidence which to our knowledge has not been assembled before, which we have carefully selected, collated, and summarized. There are some limitations. Hand-searching of reference lists for additional citations was not performed due to the large number of results obtained during data-base searching. Relevant interventions may have therefore been missed. Important insights may also have been missed by excluding studies that focused exclusively on parents of children with behavioral, emotional or mental health conditions, and we may have found additional eligible studies had we included an even broader search. Excluding interventions potentially not suited for generalist or lay worker delivery was a subjective decision. Although we made these decisions carefully as a team and only excluded interventions which required a high degree of individual tailoring by a qualified psychiatrist or psychologist, this may also have resulted in studies with meaningful and relevant information being excluded. As this was a scoping review detailed quality assessment of each study was not performed, and the results were not quantified and therefore the reported effectiveness of interventions should be interpreted with caution.

The scope of interventions to reduce parent and caregiver stress for parents of CMC has been previously reviewed [83]. Care coordination models, respite care, telemedicine, peer and emotional support, insurance and employment benefits, and health and related supports were identified as promising approaches to reduce stress. The very broad conceptualization of stress adopted within that review suggests these approaches may also contribute to improved parent health and wellbeing. In contrast, our review presented here has focused exclusively on interventions where outcomes have been directly assessed at the level of individual parents, and which could be provided at the point of routine health care. Including studies where parent outcomes have been measured directly has focused the scope of our findings to largely (but not exclusively) the domain of emotional and peer support. Our wide range of included outcomes has, however, enabled us to gain information from additional interventions that are not explicitly related to stress theory.

This is not the first review to identify the lack of consistency in outcome measures within the field. A previous scoping review of patient- and family-oriented outcomes and measures for chronic pediatric disease also reported a high number of outcomes spanning domains of general health status and quality of life, physical health and functional status, social health and relationships, mental health, and disease management and perceptions [84]. Although the high number and wide range of outcomes was perhaps to be expected given the breadth of this review and the range of parent needs, it is noteworthy that the most common outcome, anxiety, was measured in less than a third of studies, and next most common outcomes, stress, depression and coping in less than a fifth. The range of additional outcomes measured across the body of studies adds further weight to our interpretation that parents have multiple and varied needs across their child’s illness trajectory. Further research to elucidate the challenges faced by parents that are amenable to intervention within routine care settings, mechanisms of action, and related outcome measures would be helpful for future intervention development. Given the extremely limited evidence of parental involvement in all aspects of intervention development and evaluation, development of core outcomes informed by the perspectives of parents of CSHCN will also ensure that interventions measure and are assessed on outcomes relevant and meaningful to parents.

## Conclusions

The range of interventions identified by this review reveal that parents of CSHCN and CMC have significant support needs, and that there is a substantial, broad and growing evidence base for interventions to improve parent outcomes, with much scope for these to be provided by the multi-disciplinary team during routine health care contacts. Further review of the existing literature is needed to quantify the benefits for parents and assess the quality of the evidence. Further development of interventions to address issues that are relevant and meaningful to parents is needed to maximize intervention effectiveness in this context.

## Additional files

Additional file 1: Medline search. (DOCX 13 kb)

Additional file 2: Excluded studies with reasons. (DOCX 24 kb)

Additional file 3: Summary of included studies. (DOCX 61 kb)

## Data Availability

The datasets used and/or analyzed during the current study are available from the corresponding author on reasonable request.

## Abbreviations

CMC: Children with medical complexity
CSHCN: Children with special healthcare needs
PRISMA: Preferred Reporting Items for Syatematic Reviews and Meta-Analyses
RCT: Randomized Controlled Trial
TiDieR: Template for Intervention Description and Replication

## References

[1] McPherson M, Arango P, Fox H, Lauver C, McManus M, Newacheck P (1998). A new definition of children with special health care needs. Pediatrics..

[2] Cohen E, Kuo DZ, Agrawal R, Berry JG, Bhagat SK, Simon TD (2011). Children with medical complexity: an emerging population for clinical and research initiatives. Pediatrics..

[3] Berry JG. What Children with Medical Complexity, Their Families, and Healthcare Providers Deserve from an Ideal Healthcare System: Lucile Packard Foundation for Chidlren’s Health; 2015 [Briefing Paper]. Available from: https://www.lpfch.org/sites/default/files/field/publications/idealhealthcaresystem.pdf.

[4] Rolland JS, Walsh F (2006). Facilitating family resilience with childhood illness and disability. Curr Opin Pediatr.

[5] Smith J, Swallow V, Coyne I (2015). Involving parents in managing their child's long-term condition-a concept synthesis of family-centered care and partnership-in-care. J Pediatr Nurs.

[6] Pinquart M (2013). Do the parent-child relationship and parenting behaviors differ between families with a child with and without chronic illness? A meta-analysis. J Pediatr Psychol.

[7] Cousino MK, Hazen RA (2013). Parenting stress among caregivers of children with chronic illness: a systematic review. J Pediatr Psychol.

[8] Eccleston C, Fisher E, Law E, Bartlett J, Palermo TM (2015). Psychological interventions for parents of children and adolescents with chronic illness. Cochrane Database Syst Rev.

[9] Annaim A, Lassiter M, Viera AJ, Ferris M (2015). Interactive media for parental education on managing children chronic condition: a systematic review of the literature. BMC Pediatr.

[10] Arksey H, O'Malley L (2005). Scoping studies: towards a methodological framework. Int J Soc Res Methodol.

[11] JBI (2015). Joanna Briggs Institute Reviewers’ Manual: 2015 edition/supplement.

[12] Bradshaw SR, Shaw K, Bem D, Cummins C (2017). Improving health, well-being and parenting skills in parents of children with medical complexity: a scoping review protocol. BMJ Open.

[13] Hsieh HF, Shannon SE (2005). Three approaches to qualitative content analysis. Qual Health Res.

[14] Hoffmann TC, Glasziou PP, Boutron I, Milne R, Perera R, Moher D (2014). Better reporting of interventions: template for intervention description and replication (TIDieR) checklist and guide. BMJ..

[15] Nicholas DB, Keilty K (2007). An evaluation of dyadic peer support for caregiving parents of children with chronic lung disease requiring technology assistance. Soc Work Health Care.

[16] Ireys HT, Sills EM, Kolodner KB (1996). A social support intervention for parnets of children with juvenile rheumatoid athritis: results of a randomised controlled trial. J Pediatr Psychol.

[17] Barlow J, Smailagic N, Huband N, Roloff V, Bennett C (2014). Group-based parent training programmes for improving parental psychosocial health. Cochrane Database Syst Rev.

[18] Minor HG, Carlson LE, Mackenzie MJ, Zernicke K, Jones L (2006). Evaluation of a mindfulness-based stress reduction (MBSR) program for caregivers of children with chronic conditions. Soc Work Health Care.

[19] Hernandez NE, Kolb S (1998). Effects of relaxation on anxiety in primary caregivers of chronically ill children. Pediatr Nurs.

[20] Curle C, Bradford J, Thompson J, Cawthron P (2005). Users’ views of a group therapy intervention for chronically ill or disabled children and their parents: towards a meaningful assessment of therapeutic effectiveness. Spec Issue.

[21] Chaves C, Hervas G, Vazquez C (2016). Granting wishes of seriously ill children: effects on parents’ well-being. J Health Psychol.

[22] Lind Ane, Jensen Lene, Holm Birthe B (2012). Rare family days: a family empowerment programme. Orphanet Journal of Rare Diseases.

[23] Stuttard L, Beresford B, Clarke S, Beecham J, Todd S, Bromley J (2014). Riding the rapids: living with autism or disability -- an evaluation of a parenting support intervention for parents of disabled children. Res Dev Disabil.

[24] Goll-Kopka A (2009). Multi-family therapy (MFT) with families of children with developmental delays, chronic illness and disabilities: “the Frankfurt multi-family therapy model.”. Prax Kinderpsychol Kinderpsychiatr.

[25] Kieckhefer G, Trahms C, Churchill S, Kratz L, Uding N, Villareale N (2014). A randomized clinical trial of the building on family strengths program: an education program for parents of children with chronic health conditions. Matern Child Health J.

[26] Barlow J, Swaby L, Turner A (2008). Perspectives of parents and tutors on a self-management program for parents/guardians of children with long-term and life-limiting conditions: “a life raft we can sail along with.”. J Community Psychol.

[27] Dellve L (2006). Stress and well-being among parents of children with rare diseases: a prospective intervention study. J Adv Nurs.

[28] Othman A, Blunder S, Mohamad N, Hussin ZAMR, Osman ZJ (2010). Piloting a psycho-education program for parents of pediatric cancer patients in Malaysia. Psychooncology..

[29] Jerram H, Raeburn J, Stewart A (2005). The strong parents-strong children Programme: parental support in serious and chronic child illness. N Z Med J.

[30] Glazer-Waldman HR, Zimmerman JE, Landreth GL, Norton D (1992). Filial therapy: an intervention for parents of children with chronic illness. Int J Play Ther.

[31] Burke SO, Handley-Derry MH, Costello EA, Kauffmann E, Dillon MC (1997). Stress-point intervention for parents of repeatedly hospitalized children with chronic conditions. Res Nurs Health.

[32] Burke SO, Harrison MB, Kauffmann E, Wong C (2001). Effects of stress-point intervention with families of repeatedly hospitalized children. J Fam Nurs.

[33] Starks H, Doorenbos A, Lindhorst T, Bourget E, Aisenberg E, Oman N (2016). The family communication study: a randomized trial of prospective pediatric palliative care consultation, study methodology and perceptions of participation burden. Contemp Clin Trials.

[34] Hamall KM, Heard TR, Inder KJ, McGill KM, Kay-Lambkin F. The Child Illness and Resilience Program (CHiRP): a study protocol of a stepped care intervention to improve the resilience and wellbeing of families living with childhood chronic illness. BMC Psychol. 2014;2(5). 10.1186/2050-7283-2-5 PMC4416421 25945251

[35] Li Y, Wei M, Page G, Immelt S, Lu CM (2010). Effectiveness of educational interventions in children with chronic diseases and their parents. Chin J Contemp Pediatr.

[36] Sadeghi Shabestari M, Valizadeh S, Goli H (2013). The effect of massage therapy for children with asthma on maternal anxiety. Iran J Allergy Asthma Immunol.

[37] Vera MF, Cardenas SJ, Vera JG (2016). Effects of a single-session intervention on anxiety and depression in informal primary caregivers. Efectos de una Intervencion de Sesion Unica Sobre la Ansiedad y Depresion en Cuidadores Primarios Informales.

[38] Wacharasin C, Phaktoop M, Sananreangsak S (2015). Examining the usefulness of a family empowerment program guided by the illness beliefs model for families caring for a child with thalassemia. J Fam Nurs.

[39] Hackworth NJ, Matthews J, Burke K, Petrovic Z, Klein B, Northam EA (2013). Improving mental health of adolescents with type 1 diabetes: protocol for a randomized controlled trial of the nothing ventured nothing gained online adolescent and parenting support intervention. BMC Public Health.

[40] Morawska A, Mitchell A, Burgess S, Fraser J (2017). Randomized controlled trial of triple P for parents of children with asthma or eczema: effects on parenting and child behavior. J Consult Clin Psychol.

[41] Lohan A, Mitchell AE, Filus A, Sofronoff K, Morawska A. Positive parenting for healthy living (Triple P) for parents of children with type 1 diabetes: Protocol of a randomised controlled trial. BMC Pediatrics. 2016;16(1). 10.1186/s12887-016-0697-4 PMC5034659 27659518

[42] Doherty FM, Calam R, Sanders MR (2013). Positive parenting program (triple P) for families of adolescents with type 1 diabetes: a randomized controlled trial of self-directed teen triple P. J Pediatr Psychol.

[43] Rayner M, Muscara F, Dimovski A, McCarthy MC, Yamada J, Anderson VA, et al. Take A Breath: Study protocol for a randomized controlled trial of an online group intervention to reduce traumatic stress in parents of children with a life threatening illness or injury. BMC Psychiatry. 2016;16(1). 10.1186/s12888-016-0861-2 PMC4884427 27234569

[44] Wallace DP, Woodford B, Connelly M (2016). Promoting psychological flexibility in parents of adolescents with chronic pain: pilot study of an 8-week group intervention. Clin Pract Pediatr Psychol.

[45] Lindstrom C, Aman J, Anderzen-Carlsson A, Lindahl NA (2016). Group intervention for burnout in parents of chronically ill children-a small-scale study. Scand J Caring Sci.

[46] Palermo TM, Law EF, Bromberg M, Fales J, Eccleston C, Wilson AC (2016). Problem-solving skills training for parents of children with chronic pain: a pilot randomized controlled trial. Pain..

[47] Sahler OJZ, Varni JW, Fairclough DL, Butler RW, Noll RB, Dolgin MJ (2002). Problem-solving skills training for mothers of children with newly diagnosed cancer: a randomized trial. J Dev Behav Pediatr.

[48] Grey M, Jaser SS, Whittemore R, Jeon S, Lindemann E (2011). Coping skills training for parents of children with type I diabetes: 12-month outcomes. Nurs Res.

[49] Kaslow NJ, Collins MH, Rashid FL, Baskin ML, Griffith JR, Hollins L (1997). The efficacy of a pilot family psychoeducational intervention for pediatric sickle cell disease (SCD). Families, systems and health. 2000;18(4):381-404. Additional information: Kaslow NJ, Collins MH, Loundy MR, Brown F, Hollins LD, Eckman J. empirically validated family interventions for pediatric psychology: sickle cell disease as an exemplar. J Pediatr Psychol.

[50] Ridge K, Thomas S, Jackson P, Pender S, Heller S, Treasure J (2014). Diabetes-oriented learning family intervention (DOLFIN): a feasibility study evaluating an intervention for carers of young persons with type 1 diabetes. Diabet Med.

[51] Lewis MA, Hatton CL, Salas I, Leake B, Chiofalo N (1991). Impact of the Children’s epilepsy program on parents. Epilepsia..

[52] Broquet Ducret C, Verga ME, Stoky-Hess A, Verga J, Gehri M (2013). Impact of a small-group educational intervention for 4- to 12-year-old asthmatic children and their parents on the number of healthcare visits and quality of life. Arch Pediatr.

[53] Mattsson A, Agle DP (1972). Group therapy with parents of hemophiliacs: therapeutic process and observations of parental adaptation to chronic illness in children. J Am Acad Child Psychiatry.

[54] Staab D (2010). Structured educational programs for children with atopic dermatitis and their parents. Allergologie..

[55] Haberle H, Schwarz R, Mathes L (1997). Family orientated interventions of children and adolescents with cancer disease. Familienorientierte Betruung bei krebskranken Kindern und Jugendlichen.

[56] Hagglund KJ, Doyle NM, Clay DL, Frank RG, Johnson JC, Pressly TA (1996). A family retreat as a comprehensive intervention for children with arthritis and their families. Arthritis Care Res.

[57] Marteau TM, Gillespie C, Swift PG (1987). Evaluation of a weekend group for parents of children with diabetes. Diabet Med.

[58] Swallow VM, Knafl K, Santacroce S, Campbell M, Hall AG (2014). Smith T, et al. JMIR Res Protoc.

[59] Akre C, Ramelet A-S, Berchtold A, Suris J-C (2015). Educational intervention for parents of adolescents with chronic illness: a pre-post test pilot study. Int J Adolesc Med Health.

[60] Tew KL (1997). The efficacy of filial therapy with families with chronically ill children. Diss Abstr Int Sect A Humanit Soc Sci.

[61] Stewart M, Letourneau N, Masuda JR, Anderson S, McGhan S (2011). Online solutions to support needs and preferences of parents of children with asthma and allergies. J Fam Nurs.

[62] Leonard H, Slack-Smith L, Phillips T, Richardson S, D'Orsogna L, Mulroy S (2004). How can the internet help parents of children with rare neurologic disorders?. J Child Neurol.

[63] Ritchie J, Stewart M, Ellerton M-L, Thompson D, Meade D, Viscount PW (2000). Parents’ perceptions of the impact of a telephone support group intervention. J Fam Nurs.

[64] Ireys HT, Chernoff R, DeVet KA, Kim Y (2001). Maternal outcomes of a randomized controlled trial of a community-based support program for families of children with chronic illnesses. Arch Pediatr Adolesc Med.

[65] Melnyk BM (1994). Coping with unplanned childhood hospitalization: effects of informational interventions on mothers and children. Nurs Res.

[66] Melnyk BM, Alpert-Gillis LJ, Hensel PB, Cable-Beiling RC, Rubenstein JS (1997). Helping mothers cope with a critically ill child: a pilot test of the COPE intervention. Res Nurs Health.

[67] Melnyk BM, Alpert-Gillis L, Feinstein NF, Crean HF, Johnson J, Fairbanks E (2004). Creating opportunities for parent empowerment: program effects on the mental health/coping outcomes of critically ill young children and their mothers. Pediatrics..

[68] Als LC, Vickers B, Nadel S, Cooper M, Garralda ME (2014). A brief intervention to improve parent post-traumatic stress symptoms following paediatric critical illness: a pilot randomised controlled trial. Arch Dis Child.

[69] Barrera M, Hancock K, Rokeach A, Atenafu E, Cataudella D, Punnett A (2014). Does the use of the revised psychosocial assessment tool (PATrev) result in improved quality of life and reduced psychosocial risk in Canadian families with a child newly diagnosed with cancer?. Psycho-Oncology..

[70] Clarke-Pounder JP, Boss RD, Roter DL, Hutton N, Larson S, Donohue PK (2015). Communication intervention in the neonatal intensive care unit: can it backfire?. J Palliat Med.

[71] Hancock Hayley S., Pituch Ken, Uzark Karen, Bhat Priya, Fifer Carly, Silveira Maria, Yu Sunkyung, Welch Suzanne, Donohue Janet, Lowery Ray, Aiyagari Ranjit (2018). A randomised trial of early palliative care for maternal stress in infants prenatally diagnosed with single-ventricle heart disease. Cardiology in the Young.

[72] Danuser U, Estermann C, Landolt M, Rutishauser C, Kriemler S (2013). Effectiveness of an exercise intervention on healthrelated quality of life and well-being in parents from hospitalized children with severe illness: a randomized-controlled trial. Swiss Med Wkly.

[73] Tsuruta K, Kusaba H, Yamada M, Murakata T, Nakatomi R (2005). Health support program for family members with hospitalized child. Pediatr Nurs.

[74] Barlow JH, Powell LA, Gilchrist M, Fotiadou M (2008). The effectiveness of the training and support program for parents of children with disabilities: a randomized controlled trial. J Psychosom Res.

[75] Schwartz L, Drotar D (2004). Effects of written emotional disclosure on caregivers of children and adolescents with chronic illness. J Pediatr Psychol.

[76] DeMaso DR, Gonzalez-Heydrich J, Erickson JD, Grimes VP, Strohecker C (2000). The experience journal: a computer-based intervention for families facing congenital heart disease. J Am Acad Child Adolesc Psychiatry.

[77] Svavarsdottir EK. Strengths-oriented therapeutic conversations for families of children with chronic illnesses : findings from the Landspitali university hospital family nursing implementation project. J Fam Nurs. 2014;20(1):13–50. 10.1177/1074840713520345 24470558

[78] Svavarsdottir EK, Tryggvadottir GB, Sigurdardottir AO (2012). Knowledge translation in family nursing: does a short-term therapeutic conversation intervention benefit families of children and adolescents in a hospital setting? Findings from the Landspitali University Hospital family nursing implementation project. J Fam Nurs.

[79] Stabler B, North Carolina Univ CHSoM, et al. Facilitating positive psychosocial adaptation in children with cystic fibrosis by increasing family communication and problem-solving skills: a research report to the cystic fibrosis foundation. Paper presented at the Annual Meeting of the American Psychological Association 89th, Los Angeles, CA, August 24-28, 1981.

[80] Worley G, Rosenfeld LR, Lipscomb J (1991). Financial counseling for families of children with chronic disabilities. Dev Med Child Neurol.

[81] Emerson L-M, Bögels S (2017). A systemic approach to pediatric chronic health conditions: why we need to address parental stress. J Child Fam Stud.

[82] Peckham A, Spalding K, Watkins J, Bruce-Barrett C, Grasic M, Williams AP (2014). Caring for Caregivers of High Needs Children. Healthc Q.

[83] Edelstein H, Schippke J, Sheffe S, Kingsnorth S (2017). Children with medical complexity: a scoping review of interventions to support caregiver stress. Child Care Health Dev.

[84] Khangura SD, Karaceper MD, Trakadis Y, Mitchell JJ, Chakraborty P, Tingley K, et al. Scoping review of patient- and family-oriented outcomes and measures for chronic pediatric disease. BMC Pediatr. 2015;15(1). 10.1186/s12887-015-0323-x PMC4334411 25886474

