# Supplementary material for: Improving health, wellbeing and parenting skills in parents of children with special health care needs and medical complexity – a scoping review
Source: BMC Pediatr. 2019 Aug 30;19:301. doi: 10.1186/s12887-019-1648-7 (PMC6716943; doi:10.1186/s12887-019-1648-7)
Supplement: Supplementary file 1 — Medline search. (DOCX 13 kb) [file 12887_2019_1648_MOESM1_ESM.docx]

# Additional file 1 Medline search

Database: Ovid MEDLINE(R) <1946 to February Week 3 2017> Searched 23^rd^ February 2017

1 (parent$ or father$1 or mother$1 or care$ or family or families).ti. (619585)

2 exp Parents/ed, px or exp Fathers/ed, px or exp Mothers/ed, px or exp Caregivers/ed, px or exp Single Parent/ed, px (62541)

3 1 or 2 (644170)

4 ("children with special health care needs" or CSHCN).mp. (769)

5 Chronic Pain/ or Hospital, Chronic Disease/ or Chronic Disease/ (249888)

6 ((long term or chronic$ or "activity limiting" or disorder$1 or condition$1 or ill$ or sick or pain or "medical complexity") adj3 (child or children or kid$1 or toddler$1 or teen$ or minor$1 or juvenile$1 or youth$1 or adolescent$1 or p?ediatric$1 or baby or babies or neonat$)).ti,ab. (75644)

7 Health/ or Social Adjustment/ or Emotional Adjustment/ or Adaptation, Psychological/ or Quality of Life/ or Stress, Psychological/ or Resilience, Psychological/ or Self Concept/ or Self Efficacy/ (395205)

8 (health or wellbeing or adjustment or adaptation or "quality of life" or resilience or stress or cope or coping or "self efficacy" or "self concept" or anxiety or support).ti,ab. (2597852)

9 (training or education or skill$1 or program$ or intervention$1).ti,ab. (1560078)

10 7 or 8 or 9 (3722331)

11 exp child/ or pediatrics/ (1727135)

12 5 and 11 (27305)

13 4 or 6 or 12 (96819)

14 3 and 10 and 13 (7786)

15 limit 14 to humans (7716)

16 limit 15 to (clinical study or clinical trial, all or comparative study or evaluation studies or observational study or systematic reviews) (1721)

17 ("opposition$ defiance disorder" or autism or ASD or "attention deficit disorder" or ADHD or "end of life" or transition$).ti. (75897)

18 16 not 17 (1662)
