# Supplementary material for: Improving health, wellbeing and parenting skills in parents of children with special health care needs and medical complexity – a scoping review
Source: BMC Pediatr. 2019 Aug 30;19:301. doi: 10.1186/s12887-019-1648-7 (PMC6716943; doi:10.1186/s12887-019-1648-7)
Supplement: Supplementary file 2 — Excluded studies with reasons. (DOCX 24 kb) [file 12887_2019_1648_MOESM2_ESM.docx]

# Additional file 2: Excluded studies with reasons

| **Reference** | **Reason excluded** |
| --- | --- |
| Amico J, Davidhizar R. Supporting families of critically ill children. Journal of Clinical Nursing. 1994;3(4):213-218. | Eligible parent outcomes not reported |
| Anderson B, Loughlin C, Goldberg E, Laffel L. Comprehensive, family-focused outpatient care for very young children living with a chronic disease: Lessons from a program in pediatric diabetes. *Special Issue: Comprehensive care for children with chronic health conditions.* 2001;4(4):235-250.2011 | Eligible parent outcomes not reported |
| Baywater EM. Coping with a life-threatening illness: an experiment in parents' groups. *British Journal of Social Work.* 1984;14(2):117-127. | Eligible parent outcomes not reported |
| Bhana A, Mellins CA, Petersen I, et al. The VUKA family program: Piloting a family-based psychosocial intervention to promote health and mental health among HIV infected early adolescents in South Africa. AIDS Care. 2014;26(1):1-11. | Eligible parent outcomes not reported |
| Camden CP, Foley VM, Anaby DP, et al. Using an evidence-based online module to improve parents' ability to support their child with Developmental Coordination Disorder. Disability and Health Journal. 2016;9(3):406. | Eligible parent outcomes not reported |
| Carter CB. The effects of targeted vs. non-specific interventions in family therapy for teens with epilepsy and their parents. Dissertation Abstracts International: Section B: The Sciences and Engineering. 2002;62(11-B):5365. | Eligible parent outcomes not reported |
| Charepe ZB, Figueiredo MHdJS, Vieira MMdS, Neto LMVA. (Re)discovering hope in families of children with chronic disease through genogram and ecomap. Texto & Contexto Enfermagem. 2011;20(2):349-358. | Eligible parent outcomes not reported |
| Cloutier PF, Manion IG, Walker JG, Johnson SM. Emotionally focused interventions for couples with chronically ill children: a 2-year follow-up. *Journal of marital and family therapy.* 2002;28(4):391-398. | Requires specialist |
| Colville GA, Cream PR, Kerry SM. Do parents benefit from the offer of a follow-up appointment after their child's admission to intensive care?: an exploratory randomised controlled trial. Intensive and Critical Care Nursing. 2010;26(3):146-153. | Study population not eligible |
| Dawson JS. A spiritual resource of hope and healing for parents of seriously ill children. Dissertation Abstracts International Section A: Humanities and Social Sciences. 2008;69(3-A):1004. | Eligible parent outcomes not reported |
| de Oliveira IR, Nascif-Junior IA, Rocha SMM. Promoting health in families of children with type 1 diabetes mellitus. International Journal of Nursing Practice. 2010;16(2):106-111. | Eligible parent outcomes not reported |
| Distelberg B, Williams-Reade J, Tapanes D, Montgomery S, Pandit M. Evaluation of a family systems intervention for managing pediatric chronic illness: Mastering Each New Direction (MEND). Family process. 2014;53(2):194-213. | Requires specialist |
| Dodds CB, Bjornson KF, Sweeney JK, Narayanan UG. The effect of supported physical activity on parental-reported health-related quality of life in children with medical complexity. Journal of pediatric rehabilitation medicine. 2015;8(2):83-95 | Intervention does not include parents |
| Drotar D. Psychological interventions for children with chronic physical illness and their families: Toward integration of research and practice. Handbook of psychotherapies with children and families. 1999:447-461. | Book chapter |
| duTreil S, Rice J, Merritt D, Kuebler EJ. Parents Empowering Parents (PEP) Program: understanding its impact on the bleeding disorders community. Haemophilia : the official journal of the World Federation of Hemophilia. 2011;17(5):e895-900. | Eligible parent outcomes not reported |
| Emmel ML, Matsukura TS. Grupo-suporte de maes de criancas em atendimento em terapia ocupacional: Support group of mothers with children in occupational therapy. Psicologia (Universidade de Brasilia Impresso): teoria e pesquisa 1989;5(3):315-323. | Eligible parent outcomes not reported |
| Foreman T, Willis L, Goodenough B. Hospital-Based Support Groups for Parents of Seriously Unwell Children: An Example from Pediatric Oncology in Australia. Social Work with Groups. 2005;28(2):3-21. | Eligible parent outcomes not reported |
| Freda MC. Can patient education help mothers of sick children cope? MCN The American journal of maternal child nursing. 1998;23(1):52. | Summary of intervention already included |
| Gewirtz A, Gossart-Walker S. Home-based treatment for children and families affected by HIV and AIDS: Dealing with stigma, secrecy, disclosure, and loss. Special Issue: Children and Adolescents Affected by HIV/AIDS: A Mental Health Challenge. 2000;9(2):313-330. | Eligible parent outcomes not reported |
| Gibb C, Lawton S, Roberts A. Supporting the parents of children with atopic eczema. British Journal of Nursing. 2005;14(13):693-696. | Eligible parent outcomes not reported |
| Gordon J. An evidence-based approach for supporting parents experiencing chronic sorrow. Pediatric nursing. 2009;35(2):115-119. | Eligible parent outcomes not reported |
| Gupta V, Prescott H. "That must be so hard"- Examining the impact of children's palliative care services on the psychological well-being of parents. Clinical Child Psychology and Psychiatry. 2013;18(1):91-99. | Not a defined intervention or cohort |
| Harris M. Program: Empowering Parents of Children With Special Health Care Needs: Harnessing the Power of the Internet. Health Education & Behavior. 2004;31(6):664-666. | Eligible parent outcomes not reported |
| Hartman AF, Radin MB, McConnell B. Parent-to-parent support: A critical component of health services for families. Issues in Comprehensive Pediatric Nursing: Special Issue: Family-centered, community-based, coordinated care for children with special health care needs. 1992;15(1):55-67. | Not a defined intervention or cohort |
| Hashemi F, Shokrpour N. The impact of education regarding the needs of pediatric leukemia patients' siblings on the parents' knowledge and practice. The Health Care Manager. 2010;29(1):75-79. | Eligible parent outcomes not reported |
| Heap K. Short term groupwork in the treatment of chronic sorrow: a Norwegian experience. Groupwork. 1988;1(3):197-214. | Eligible parent outcomes not reported |
| Herbert LJ, Sweenie R, Kelly KP, Holmes C, Streisand R. Using qualitative methods to evaluate a family behavioral intervention for type 1 diabetes. Journal of Pediatric Health Care. 2014;28(5):376-385. | Eligible parent outcomes not reported |
| Hinrichsen GA, et al. Illness and Prevention: Self-Help Groups for Families Faced with Scoliosis. 1981. | Not a defined intervention or cohort |
| Holt C, Kotal-Lee RB, Winston B, Deringer AC, Nodvin J, Howett M. The use of home-based caregiver assessment to improve children's health: A pilot project. International Journal on Disability and Human Development. 2013;12(4):427-431. | Eligible parent outcomes not reported |
| Hopia H, Paavilainen E, Tomlinson PS, Astedt-Kurki P. Child in hospital: family experiences and expectations of how nurses can promote family health. Journal of Clinical Nursing. 2005;14(2):212-222. | Not a defined intervention or cohort |
| Kaslow NJ, Brown F. Culturally sensitive family interventions for chronically ill youth: Sickle cell disease as an example. Special Issue: Child and Adolescent Illness and Treatment. 1995;13(2):201-213. | Eligible parent outcomes not reported |
| Kaslow NJ, Collins MH, Loundy MR, Brown F, Hollins LD, Eckman J. Empirically validated family interventions for pediatric psychology: sickle cell disease as an exemplar. Journal of pediatric psychology. 1997;22(2):213-227. | Interrim report, full results included |
| Koliopoulou V, Pergantou H, Xafaki P, Platokouki H. Concerns of parents of haemophilic children related to the disease and its treatment: Experience from a support group. Haemophilia. 2014;20:52. | Eligible parent outcomes not reported |
| Kratz L, Uding N, Trahms CM, Villareale N, Kieckhefer GM. Managing childhood chronic illness: parent perspectives and implications for parent-provider relationships. Families, Systems & Health: The Journal of Collaborative Family HealthCare. 2009;27(4):303-313. | Eligible parent outcomes not reported |
| Liberman DB, Song E, Radbill LM, Pham PK, Derrington SF. Early introduction of palliative care and advanced care planning for children with complex chronic medical conditions: A pilot study. Child: Care, Health and Development. 2016;42(3):439-449. | Eligible parent outcomes not reported |
| Martin S, Wolters PL, Toledo-Tamula MA, et al. Acceptance and commitment therapy in youth with neurofibromatosis type 1 (NF1) and chronic pain and their parents: A pilot study of feasibility and preliminary efficacy. American Journal of Medical Genetics, Part A. 2016;170(6):1462-1470. | Eligible parent outcomes not reported |
| Massimo LM. Relationship between parents and sick children: Difficulties and possibilities regarding understanding. Hauppauge, NY, US: Nova Science Publishers US; 2006. | Book chapter |
| Masuda A, Cohen LL, Wicksell RK, Kemani MK, Johnson A. A case study: Acceptance and commitment therapy for pediatric sickle cell disease. Journal of pediatric psychology. 2011;36(4):398-408. | Requires specialist |
| McCarthy MJ, Herbert R, Brimacombe M, Hansen J, Wong D, Zelman M. Empowering parents through asthma education. Pediatric nursing. 2002;28(5):465-473. | Eligible parent outcomes not reported |
| McMenamy JM, Perrin EC. Filling the GAPS: description and evaluation of a primary care intervention for children with chronic health conditions. Ambulatory pediatrics : the official journal of the Ambulatory Pediatric Association. 2004;4(3):249-256. | Eligible parent outcomes not reported |
| Mollison H, Helen Abrams H, Barrett E, Hamilton J, Maas E, Azmy A. Group meetings for parents in a children's burns unit. Scottish Medical Journal. 1983;28(2):168-171. | Eligible parent outcomes not reported |
| Noeker M, Petermann F, Bode U. Family counseling in childhood cancer: Conceptualization and empirical results. Theoretical and applied aspects of health psychology. 1990:241-253. | Book chapter |
| O'Connell MA. Maternal sensitivity in a high-risk, African American preterm sample: An ecological approach to predictors and effects of intervention. Dissertation Abstracts International: Section B: The Sciences and Engineering. 2008;68(10-B):7003. | Not available |
| Palermo TM, Wilson AC, Peters M, Lewandowski A, Somhegyi H. Randomized controlled trial of an Internet-delivered family cognitive-behavioral therapy intervention for children and adolescents with chronic pain. Pain. 2009;146(1-2):205-213. | Eligible parent outcomes not reported |
| Palermo TM, Law EF, Fales J, Bromberg MH, Jessen-Fiddick T, Tai G. Internet-delivered cognitive-behavioral treatment for adolescents with chronic pain and their parents: A randomized controlled multicenter trial. Pain. 2015;157(1):174-185. | Eligible parent outcomes not reported |
| Peckham A, Spalding K, Watkins J, Bruce-Barrett C, Grasic M, Williams AP. Caring for Caregivers of High-Needs Children. Healthcare Quarterly. 2014;17(3):30-35. | Not a defined intervention or cohort |
| Pickett K, Frampton G, Loveman E. Education to improve quality of life of people with chronic inflammatory skin conditions: a systematic review of the evidence. British Journal of Dermatology. 2016;174(6):1228-1241. | Review |
| Piske F, Azevedo LA, Marcon C, Barbosa Oliveira LD. Support group for caregivers of children admitted to a pediatric unit. Grupo de apoio para acompanhantes de criancas internadas em uma unidade pediatrica. 2013;15(1):35-49. | Eligible parent outcomes not reported |
| Plumridge G, Metcalfe A, Coad J, Gill P. The role of support groups in facilitating families in coping with a genetic condition and in discussion of genetic risk information. Health Expectations. 2012;15(3):255-266. | Not a defined intervention or cohort |
| Rau J, May TW, Pfafflin M, Heubrock D, Petermann F. Education of children with epilepsy and their parents by the modular education program epilepsy for families (FAMOSES) - Results of an evaluation study. Rehabilitation. 2006;45(1):27-39. | Eligible parent outcomes not reported |
| Raundalen M, Dyregrov A. Psychological intervention in families with a seriously ill child: Therapeutic means: II. Psykologisk intervensjon i familier med alvorlig syke barn: Terapeutiske virkemidler Del II. 1983;20(2):61-69. | Eligible parent outcomes not reported |
| Rennick JE, Lambert S, Childerhose J, Campbell-Yeo M, Filion F, Johnston CC. Mothers' experiences of a Touch and Talk nursing intervention to optimise pain management in the PICU: A qualitative descriptive study. Intensive and Critical Care Nursing. 2011;27(3):151-157. | Eligible parent outcomes not reported |
| Reyhani T, Pour ZS, Heidarzadeh M, Mousavi SM, Mazloom SR. Investigating the effects of spiritual self-care training on psychological stress of mothers with preterm infants admitted in neonatal intensive care unit. Iranian Journal of Obstetrics, Gynecology and Infertility. 2014;17(97):18-27. | Study population not eligible |
| Ring EM. United States of America: 53796: maternal and child health: report on a review of maternal and child health programs and projects, with special reference to the needs of those at risk from low income and children with handicapping conditions, 9 September-20 October 1973. 1973. | Not available |
| Rios-Serrano GE. A treatment plan for caregivers of patients diagnosed with Infantile, Late Infantile or Juvenile Neuronal Ceroid Lipofuscinosis. Dissertation Abstracts International: Section B: The Sciences and Engineering. 2004;64(8-B):4059. | Not available |
| Saks JK. Adolescents with physical disabilities and their families: The impact of a short-term, multiple-family discussion group. Dissertation Abstracts International: Section B: The Sciences and Engineering. 1999;60(5-B):2365. | Not available |
| Schwartz L, Drotar D. Linguistic analysis of written narratives of caregivers of children and adolescents with chronic illness: Cognitive and emotional processes and physical and psychological health outcomes. Journal of Clinical Psychology in Medical Settings. 2004;11(4):291-301. | Eligible parent outcomes not reported |
| Seliner B, Wattinger A, Spirig R. Erfahrungen und Bedurfnisse von Eltern hospitalisierter Kinder mit Behinderung und den in der Betreuung verantwortlichen medizinischen Fachpersonen - Eine systematische Review.[Experiences and needs of parents of hospitalised children with disabilities and the health professionals responsible for the child's health-care - A systematic review]. Pflege - Die wissenschaftliche Zeitschrift für Pflegeberufe. 2015;28(5):263-276. | Review |
| Shields CG, Finley MA, Chawla N, et al. Couple and Family Interventions in Health Problems. Journal of Marital and Family Therapy 2012;38(1):265-80. | Review |
| Smith, A. B., G. C. Hefley, and K. J. Anand. "Parent Bed Spaces in the Picu: Effect on Parental Stress." Pediatric nursing 33, no. 3 (2007): 215-21. | Study population not eligible |
| Psychosocial support intervention for HIV-affected families in Haiti: Implications for programs and policies for orphans and vulnerable children | Not feasible for routine care |
| Sori CF, Blank NM. Counseling Children and Families Experiencing Serious Illness. New York, NY, US: Routledge/Taylor & Francis Group | Book chapter |
| Sridharan K, Sivaramakrishnan G. Therapeutic clowns in pediatrics: a systematic review and meta-analysis of randomized controlled trials. European Journal of Pediatrics. 2016;175(10):1353-1360 | Review |
| Staab D. Educational Measures for children with neurodermatitis and their parents. Monatsschrift fur Kinderheilkunde. 2001;149(6):560-564.1. | Review |
| Stewart MJ, Reutter L. Fostering partnerships between peers and professionals. The Canadian journal of nursing research = Revue canadienne de recherche en sciences infirmieres. 2001;33(1):97-116. | Eligible parent outcomes not reported |
| Stinson JN, McGrath PJ, Hodnett ED, et al. An internet-based self-management program with telephone support for adolescents with arthritis: A pilot randomized controlled trial. Journal of Rheumatology. 2010;37(9):1944-1952. | Eligible parent outcomes not reported |
| Szczepanski R, Seiffert S. Education of chronically ill children, adolescents and their families as well as other caregivers: status and prospects. Kinderkrankenschwester : Organ der Sektion Kinderkrankenpflege / Deutsche Gesellschaft fur Sozialpadiatrie und Deutsche Gesellschaft fur Kinderheilkunde. 2016;35(1):12-17. | Review |
| Tieffenberg JA, Wood EI, Alonso A, Tossutti MS, Vicente MF. A randomized field trial of ACINDES: A child-centered training model for children with chronic illnesses (asthma and epilepsy). Journal of Urban Health. 2000;77(2):280-397. | Not feasible for routine care |
| Toly VBPRNC, Blanchette JEBSNRN, Musil CMPRNF, Zauszniewski JAPRNBCF. Journaling as reinforcement for the resourcefulness training intervention in mothers of technology-dependent children. Applied Nursing Research : ANR. 2016;32:269. | Eligible parent outcomes not reported |
| Von Schlippe A, Theiling S, Lob-Corzilius T, Szczepanski R. The "Luftikurs:" Innovative family focused training of children with asthma in Germany. Families, Systems and Health. 2001;19(3):263-284. | Eligible parent outcomes not reported |
| Walker JG. A marital intervention program for couples with chronically ill children. Dissertation Abstracts International: Section B: The Sciences and Engineering. 2008;68(7-B):4851. | Requires specialist |
| Walker JG, Johnson S, Manion I, Cloutier P. Emotionally focused marital intervention for couples with chronically ill children. Journal of consulting and clinical psychology. 1996;64(5):1029-1036. | Requires specialist |
| Wiener L, Riekert K, Pizzo PA, Siegel K, Battles H. Exploratory study of residents' perceptions of a housing facility for pediatric patients and family members. Journal of Psychosocial Oncology. 1996;14(3):69-80. | Not feasible for routine care |
| Williams PD, Piamajariyakul U, Kataoka-Yahiro M, et al. Effects of an intervention for Hawaiian American siblings and caregivers of children with chronic illness or disability: a pilot study. UPNAAI Nursing Journal. 2009;5(1):44-46. | Not available |
