# Supplementary material for: Improving health, wellbeing and parenting skills in parents of children with special health care needs and medical complexity – a scoping review
Source: BMC Pediatr. 2019 Aug 30;19:301. doi: 10.1186/s12887-019-1648-7 (PMC6716943; doi:10.1186/s12887-019-1648-7)
Supplement: Supplementary file 3 — Summary of included studies. (DOCX 61 kb) [file 12887_2019_1648_MOESM3_ESM.docx]

# Additional file 3: Summary of included studies

| **ID No** | **Author / Year** | **Study characteristics (1-8)** | **Intervention (9-14)** | **Parental targets and measures (15, 17)** | **Parent outcomes** |
| --- | --- | --- | --- | --- | --- |
|  | Akre  2015 | **(1)** Pre-test / post-test feasibility study **(2)** Non-categorical chronic conditions requiring continuous medical management for at least the last year **(3)** Switzerland, **(4)** Any parent including step-parents if they took care of the child regularly, one parent per child **(5)** 10 – 14 years, **(6)** Group sessions, **(7)** 43, **(8)** 4-6 months | **(9)** Participatory training, **(10)** 4 X 2 hour face-to-face modules **(11)** Not mentioned, **(12)** 5, **(13)** Participatory training (UNICEF 1993) and Social Cognitive Theory (Bandura 1986 and Glanz and Bishop 2010) **(14)** Behavioral and cognitive approach, explicitly stated | **(15)** To help parents to assist their child with chronic illness to become autonomous, **(16)** Parent coping; parent perceptions of bonding; parenting stress, **(17)** Parent child shared management tool (PCSMT); Self-efficacy for Managing Chronic Disease Scale (SMCDS); Family Crisis Oriented Personal Evaluation Scale (F-COPES); Parental Bonding Instrument–Brief Current form (PBI-BC); Parental Stress Scale (PSS) | Improvement trends appeared for shared management, parent protection, and self-efficacy, and worsening trends appeared for coping skills, parental perception of child vulnerability, and parental stress. As a pilot this study is underpowered, none of the differences were statistically significant, and results should be interpreted with caution. |
|  | Als  2014 | **(1)** Pilot / feasibility RCT (abstract only), **(2)** Children admitted to PICU, **(3)** UK, **(4)** Parents, **(5)** 4-16 years, **(6)** Booklet and telephone call, **(7)** 23, **(8)** median follow up 5 months post discharge | **(9)** Post-discharge support, **(10)** Not clear , **(11)** Not mentioned, **(12)** 2, **(13)** No theory mentioned, **(14)** Psychoeducation, | **(15)** To improve parent post-traumatic stress symptoms following childhood critical illness, **(16)** Stress and PTSD, **(17)** Parental Stressor Scale: PICU (PSS-PICU); Impact of Events Scale to measure PTSS (IES) | Based on very small numbers parents in the intervention group reported fewer post-traumatic stress symptoms and fewer were at risk for clinical disorder than in the control group. However these results were not statistically significant, this study was underpowered and should be interpreted with caution. |
|  | Barlow  2008 | **(1)** Qualitative Evaluation , **(2)** Any long-term or life-limiting condition in the community, **(3)** UK, **(4)** Mothers or female relative of child, **(5)** Not specified, **(6)** Group, **(7)** 15, **(8)** To end of course | **(9)** Community based Supporting Parenting Programme (SPP), based on the Chronic Disease Self-Management course (Lorig, Sobel, Stewart, Brown, Bandura, Ritter, et al., 1999), **(10)** 15 hours in six weekly sessions, **(11)** Peers, **(12)** 8 **(13)** Chronic Disease Self-Management (Lorig, Sobel, Stewart, Brown, Bandura, Ritter, et al., 1999), and self-efficacy theory (Bandura 1977) **(14)** Behavioral cognitive approach, with explicitly referenced self-efficacy theory | **(15)** To assist parents caring for children with long-term or life-limiting conditions through support and cognitive behavioral techniques, **(16)** Parent confidence in ability to self-manage their own needs as carers for CSHCN, **(17)** Assessed qualitatively | Parents valued meeting similar others, felt less isolated, more positive, motivated, and more calm; some had found the ‘‘real me again.’’ Parents were anxious of being cast adrift at the end of the program. SPP provided a positive group experience. In the future, parents/carers should be consulted prior to attending and throughout the program to ensure it is responsive and relevant to their needs. |
|  | Barlow 2008b | **(1)** RCT, **(2)** Disabilities, **(3)** UK, **(4)** Parents, **(5)** Not specified, **(6)** 1-2-1 consultation and group sessions, **(7)** 196, **(8)** 4 months | **(9)** Parent to child massage, **(10)** 8 hours, **(11)** Qualified massage therapists **(12)** 5, **(13)** Self-efficacy (no formal theory referenced), **(14)** | **(15)** To equip parents of children with disabilities with a simple massage skill for use with their children in the home environment, **(16)** Self-efficacy, **(17)** Generalized Self-Efficacy Scale; Parent's Self-Efficacy Scale; Hospital Anxiety and Depression Scale; Perceived Stress Scales; Satisfaction with Life Scale; visual analogue scale to measure perceived health status | The intervention demonstrated statistically significant positive effects on parental self-efficacy (PSE) for managing children’s psychosocial well-being and depressed mood. There were trends toward improvement on parental satisfaction with life, global health, and parental ratings of children’s sleeping and mobility. Effect sizes were small. Levels of anxiety, depression, and perceived stress were all higher than published norms. |
|  | Barrera  2014 | **(1)** Pilot RCT , **(2)** Hospitalized children within 2 -4 weeks of a cancer diagnosis who are expected to survive over 6 months, **(3)** Canada, **(4)** Parents, **(5)** Not specified, **(6)** Parent fills out self-assessment, summary placed in child medical notes, **(7)** 67, **(8)** 6 months | **(9)** Validated psychological self-assessment summary shared with medical team, **(10)** < 1 hour to complete self-assessment, **(11)** Hospital staff, **(12)** 8, **(13)** Pediatric Psychosocial Preventive Health Model (PPPH) (Kazak 2003), **(14)** Eclectic approach referencing social ecology but dependent on referral to interventions from multiple theoretical perspectives to achieve outcomes | **(15)** To reduce psychosocial risk, **(16)** Psychosocial distress, psychosocial risk, early identification and intervention, minimal intervention, **(17)** PATrev (Psychosocial Assessment Tool) (PATrev); State Trait Anxiety Inventory - state scale only (STAI-state) | Compared to the control group, participants in the experimental group had significantly reduced targeted and clinical risk 6 months post-diagnosis. Anxiety scores reduced across the sample over time and the time effect was likely due to a clinical reduction in anxiety symptoms in the experimental group. This is a small pilot with no reported sample size calculation and results should be interpreted with caution. |
|  | Broquet Ducret 2013  French | **(1)** Observational before / after study , **(2)** Asthma, **(3)** Switzerland, **(4)** Parents, **(5)** Any, **(6)** Group sessions, **(7)** 33, **(8)** 6 months | **(9)** Therapeutic education, **(10)** 4 hours, **(11)** multidisciplinary team, **(12)** 6, **(13)** None mentioned, **(14)** | **(15)** To improve patients’ adherence to treatment and help them manage the disease on a day-to-day basis and improve parents’ quality of life, **(16)** Quality of Life, **(17)** Pediatric Asthma Caregiver's Quality of Life | Authors state that parents’ quality of life improved significantly in all domains (emotional, activities). Interpret with caution as based on a small sample size and significance was set by authors at 0.7%. |
|  | Burke  1997 | **(1)** RCT **(2)** Elective hospital admission for chronic or physically disabling condition (typically relapsing) of at least 8 months duration, **(3)** Canada, **(4)** Parents, **(5)** 1-17, **(6)** Home based, delivered individually, **(7)** Parents of 50 children, **(8)** 3 months | **(9)** Stress-point preparation, **(10)** Variable, beginning 2 weeks before hospitalization and ending 2 weeks after discharge, **(11)** Masters educated nurse, **(12)** 7, **(13)** Stress-point intervention **(14)** Eclectic mix of behavioral /cognitive and systems approaches implied through description of intervention in terms of behavioral / cognitive as well as relationship with staff (system) elements | **(15)** To ameliorate negative effects of repeated hospitalizations by decreasing parental distress and increasing parental coping, **(16)** Stress and coping, **(17)** Parent state anxiety (STAI), Feetham Family Functioning Survey (FFFS), Coping Health Inventory for Parents (CHIP) | The intervention group was reported significantly better family functioning and coping than the control group after hospitalization at 3 months, but not 2 weeks. |
|  | Burke 2001 | **(1)** Cluster RCT, analyzed at the level of individual parents, **(2)** Elective hospital re-admission for any condition, **(3)** Canada, **(4)** Parents, **(5)** Up to 15, **(6)** Clinic or sometimes home based, individual, **(7)** Parents of 139 children of whom 115 completed the study, **(8)** 3 months | **(9)** Stress point preparation, **(10)** Variable, **(11)** Experienced nurse, **(12)** 7, **(13)** Stress-point intervention, **(14)** Eclectic mix of behavioral /cognitive and systems approaches implied through description of intervention in terms of behavioral / cognitive as well as relationship with staff (system) elements | **(15)** To decrease distress and improve child and family functioning in the context of repeated hospitalizations, **(16)** Stress and coping, **(17)** Feetham Family Functioning Survey (FFFS), Coping Health Inventory for Parents (CHIP). | SPIN parents were more satisfied with family functioning and had better parental coping after hospitalization than parents who received usual care. However this should be interpreted with caution, it is a cluster randomized trial but data analysis appears to be at individual level |
|  | Chaves  2016 | **(1)** RCT, **(2)** Children recruited through the hospital, includes inpatients and outpatients, **(3)** Spain, **(4)** Parents, **(5)** 5-18 years, **(6)**, Collaboration between parents and doctors, **(7)** 124, **(8)** 3 weeks | **(9)** Wish granting **(10)** Varies according to the nature of the wish granted, **(11)** Collaboration between parents, doctors and wish granting foundation, **(12)** 3, **(13)** Hedonic and eudemonic theory (Ryan and Deci, 2001; Waterman, 2008), **(14)** Behavioral / cognitive approach associated with self-determination theory | **(15)** To promote wellbeing in parents of children with life-threatening illnesses through granting the child’s wish, **(16)** Reduced negative emotions, increased positive emotions, and improved non-hedonic dimensions of positive functioning, **(17)** Positive and Negative Emotional Style Scale (PNES); Benefit Finding Scale for Children (modified for parents) (BFSC); Beliefs in Benevolence of World Scale (BBWS); Life Orientation Test (LOT); Values in Action Inventory of Strengths (VIA-IS). | The intervention group showed higher levels of positive emotions and beliefs in a benevolent world than control group. Mothers also showed higher benefit finding, gratitude, and love than those in the control group. Wish granting did not reduce negative emotions. |
|  | Clarke-Pounder 2015 | **(1)** RCT, **(2)** NICU inpatient and expected stay greater than 14 days, and high risk for morbidity and mortality **(3)** US, **(4)** Parents, **(5)** < 8 days, **(6)** 1-2-1 interview and ward rounds, **(7)** 19, **(8)** 2 weeks | **(9)** Palliative Care Group Decision Making Tool (DMT) results shared with clinical team, **(10)** Variable, **(11)** Interview, unclear. Ward rounds lead by primary NICU provider. **(12)** 4, **(13)** None mentioned, **(14)** Implied cognitive / behavioral approach, expected to improve parent outcomes indirectly by changing health care professional behavior | **(15)** To decrease parental anxiety and increase satisfaction with care, **(16)** Stress, coping, health and competence., **(17)** STAI (state only); Family Inventory of Needs - Pediatrics; N-DTM specific survey | Based on a very small sample the control group showed non-significant decreased anxiety over time, the intervention group reported lower satisfaction with care specifically in regard to communication. |
|  | Curle  2005 | **(1)** Qualitative Evaluation **(2)** Children with any chronic condition or disability in the community attending a children’ support group, the ‘Terrific Tuesday Group’, **(3)** UK, **(4)** Parents, **(5)** 5-12 years, **(6)** Group sessions, **(7)** 27 parents from 11 out of 17 eligible families, **(8)** unclear | **(9)** Narrative therapy, **(10)** 6-8 sessions, length not specified, **(11)** Multidisciplinary professional team, **(12)** 6, **(13)** Stress and coping model of child adjustment to pediatric chronic physical disorders (Wallander and Varni 1998), **(14)** An integrated approach combining behavioral / cognitive and systems theories. | **(15)** To reduce feelings of isolation and facilitate coping **(16)** Coping, adaptation and social support, **(17)** Qualitatively evaluated using a grounded theory approach. | The benefits parents described related to their perceived reasons for attending even if these were different from the therapist’s overall aims. There was evidence of increased social support and range of coping responses. |
|  | Danuser  2013 | **(1)** RCT, abstract only, **(2)** Hospitalized children with severe illness, **(3)** Switzerland, **(4)** Parents, **(5)** Any, **(6)** Group, **(7)** 41, **(8)** follow-up not mentioned | **(9)** Exercise, **(10)** Varies, **(11)** Not stated, **(12)** 3, **(13)** No theory mentioned **(14)** Unknown theoretical approach | **(15)** To improve acute well-being and quality of life (QoL) in parents of children with severe illness while they are hospitalized, **(16)** Self-reported health, mental health, **(17)** 36-Item Short Form Survey (measures quality of life) (SF-36), Symptom Checklist-27-plus (measures mental health) (SCL-27+), Zerssen BfS/BfS' self-rating mood scale (Bf-S). | There was a significant positive effect on the mental health summary score but groups did not differ for the physical summary score or global scores. Acute well-being improved consistently in response of the exercise lessons. However there were high drop-out and non-participation rates. |
|  | Dellve  2006 | **(1)** Observational, **(2)** Rare diseases, outpatients, **(3)** Sweden, **(4)** Parents, **(5)** Any, **(6)** Group sessions, **(7)** 138 families, **(8)** 12 months | **(9)** Group education, **(10)** > 25 hours, **(11)** ‘Specialists’, **(12)** 6, **(13)** Stress, coping, empowerment and competence, **(14)** Behavioral / cognitive approach implied as intervention focusses on developing competence with child's disability therefore primarily behavioral | **(15)** To empower parents to manage their child’s rare disability, **(16)** Parental competence, **(17)** Swedish version of Parenting Stress Index (SPSQ); Ladder of Life Instrument, measures wellbeing (LLI); Interview Schedule for Supportive Interactions (ISSI);Researcher developed coping question. | Fathers’ stress related to perceived incompetence, strain among full time working parents decreased after the intervention, perceived knowledge, active coping, and mothers’ perceived social support were all improved at follow-up. Factors related to parents’ overall life satisfaction changed from being more related to internal demands (perceived strain, incompetence and social isolation) to other conditions, such as problems related to spouse, paid work and social network. However a lot of variables are reported, with no identified primary outcome, and this needs to be interpreted with caution. |
|  | DeMaso  2000 | **(1)** Mixed Methods Evaluation, **(2)** Hospitalized children with CHD, **(3)** US, **(4)** Mothers, **(5)** Any, **(6)** Computer application, **(7)** 9 (plus one adult female patient age 25 years) (Phase 1), 40 (Phase 2) , **(8)** 2-4 weeks | **(9)** On-line experience journal, **(10)** > 30 minutes, **(11)** N/A, **(12)** 6, **(13)** Preventative intervention, medical crisis counselling, narrative therapy, computer utilization, **(14)** | **(15)** To facilitate healthy coping of children with congenital heart disease and their families, **(16)** Coping, **(17)** Satisfaction, potential hurtfulness, safety and feasibility of the intervention, coping, attitude change, illness related concerns, and family functioning (all researcher developed questions). | The Experience Journal was safe and useful for decreasing social isolation, increasing understandings of familial feelings about cardiac illness and fostering positive reactions in mothers. |
|  | Doherty 2013 | **(1)** RCT, **(2)** IDDM Type 1, **(3)** UK, **(4)** Parents, **(5)** 11-17, **(6)** Workbook and tip sheet, **(7)** 90, **(8)** 10 weeks (to end of course) | **(9)** Self-directed Teen Triple P (Level 4 Triple P), **(10)** 10 hours, **(11)** N/A, **(12)** 8, **(13)** Based on social learning principles, using a public health model, **(14)** Takes an explicitly behavioral and cognitive approach based on social learning theory | **(15)** To reduce diabetes related family conflict and reduce parental stress, **(16)** Parenting conflict; stress, **(17)** Diabetes Family Conflict Scale (DFCS); Pediatric Inventory for Parents (measures parenting stress) (PIP); Eyberg Child Behavior Inventory (parental perceptions of child behavior) (ECBI); Parenting Scale (parenting style / disciplining strategies) (PS); Parenting Sense of Competence Scale (PSOC). | Significantly improved diabetes-related conflict, but not parental stress, compared with usual care. |
|  | Glazer-Waldman  1992 | (1)Observational before / after study, (2) Hospitalized any condition, (3) US, (4) Parents, (5) 4.5 – 10 years, (6) Group sessions, (7) 6, (8) Up to last session | **(9)** Filial therapy, **(10)** 20 hours, **(11)** Psychologist trained in filial therapy and a specialist social worker, **(12)** 5, **(13)** Filial therapy, an extension of play therapy, **(14)** Behavioral and cognitive approach, explicitly based on play and filial therapy. | **(15)** To teach parents the fundamentals of play therapy, **(16)** Optimal relationship between parent and child, parental anxiety and parent acceptance of child, **(17)** State Trait Anxiety Inventory (STAI), Parent Acceptance of their child (unpublished tool). | Parental anxiety and acceptance were unchanged. The match between parent and child reports of child anxiety improved indicating that parents were better able to judge their child’s anxiety. Qualitative data indicated that the parents believed that the course had a positive impact on their relationships with their children. |
|  | Goll-Kopa 2009  German | **(1)** 1 arm evaluation, after only, **(2)** No specific eligibility criteria, included developmental delays, chronic illness, disabilities, **(3)** Germany, **(4)** Parents, **(5)** Any, **(6)** 3 day residential group seminar, **(7)** 51 family members, **(8)** Not stated | **(9)** Multi-family therapy, **(10)** 17 hours, **(11)** Multi-disciplinary team **(12)** 6, **(13)** Social Pediatrics, Parenting competencies (German = Schneewind) **(14)** | **(15)** To support families of affected children in their emotional coping processes, **(16)** To improve parenting competence and ability to deal with multiple and fast changing everyday challenges, **(17)** Open questions on paper and online and focus groups to explore family members’ perception of the intervention including what works for them and what contributes to positive change. | Identified 9 beneficial factors: group solidarity, reflecting on own situation in comparison with others, hearing examples of positive change, strengthening self-efficacy, increasing confidence interacting with ‘experts’, networking, realistic weekend format away from the home situation, access to ‘nature’. |
|  | Grey 2011  RCT | **(1)** Data combined from 2 separate RCTs, **(2)** Type 1 Diabetes diagnosed at least 6 months previously, **(3)** US, **(4)** Parents, **(5)** One trial included children under 8 years old, one included 8-12 year olds, **(6)** Group sessions, **(7)** 181 families, **(8)** 12 months | **(9)** Coping skills training v. group education, **(10)** 9 hours, **(11)** Marriage and family therapist or clinical psychologist, **(12)** 6, **(13)** Social cognitive theory (Bandura) and framework for adaptation to chronic illness in childhood (Grey and Thurber, 1991), **(14)** | **(15)** To improve parental coping, family functioning and parental QOL, **(16)** Coping, family functioning, quality of life, **(17)** Issues in Coping With IDDM-Parent Scale (IC-IDDM-P); The Center for Epidemiologic Studies Depression Scale (CES-D); The Diabetes Responsibility and Conflict Scale (DRCS); The Parents Diabetes Quality of Life Questionnaire (PDQOL). | There were no significant treatment effects 12 months post-intervention but parents in both groups reported improved coping, less responsibility for treatment management, and improved quality of life. |
|  | Haberle 1997 German | **(1)** Non randomized CT, **(2)** Any cancer, **(3)** Germany, **(4)** Families including unwell child and siblings, and including palliative and after death in exceptional cases **(5)** Eligibility not specified, **(6)** 4 week rehabilitation residential, **(7)** 104, 24 with follow-up, **(8)** 12 – 16 weeks | **(9)** Residential rehabilitation, **(10)** >100, **(11)** Multi-professional team (medical, psychological& social), **(12)** 6, **(13)** Multidisciplinary bio-psycho-social approach, **(14)** 6 | **(15)** To improve psycho-social wellbeing and achieve physical, psychological and social rehabilitation for the whole family, **(16)** Stress, physical and psychological state, family interactions, **(17)** Semi structured interview analyzed using content analysis, validated scales to measure depression, fear, physical and psychological symptoms, Impact of Events scale, family function questionnaire | Improvement of physical and psychosocial wellbeing was demonstrated in quantitative and qualitative findings and high satisfaction. |
|  | Hackworth 2013 | **(1)** RCT protocol, **(2)** Type 1 diabetes, **(3)** Australia, **(4)** Parents (one per child), **(5)** 13- 18, **(6)** On-line, **(7)** To aim for 120, **(8)** 6 months | **(9)** Nothing Ventured Nothing Gained intervention (NVNG), an online adolescent and parenting intervention, **(10)** > 5 sessions for adolescents, 6 sessions for parents, over 6 weeks, **(11)** N/A, **(12)** 7, **(13)** CBT principles. The intervention is based on the ABCD parenting young adolescents program, a group-based parenting program that combines a behavioral family intervention approach with acceptance-based strategies, **(14)** | **(15)** Primary outcome to improve adolescent mental health, secondary outcomes to improve physical and psychological health of adolescents and their parents, **(16)** Adolescent behavior, parental well-being, and positive parenting practices, **(17)** Parent outcomes will be secondary outcomes including: Depression Anxiety Stress Scale (DASS); Fatigue assessment scale (FAS); Parenting Sense of Competence Scale (PSoC); Parent Experience of Child Illness Scale (PECI); Family problem solving communication index (FPSCI); Family Management Measure (FaMM); Diabetes Family Conflict Scale (DFCS); Diabetes family responsibility questionnaire (DFRQ). | N/A as protocol |
|  | Hagglund  1996 | **(1)** 1 arm evaluation, **(2)** Juvenile Rheumatoid Arthritis, outpatients, **(3)** US, **(4)** Caregivers, **(5)** 3-17 years, **(6)** Family residential retreat, **(7)** 39, **(8)** 6 months | **(9)** 3 days of multidisciplinary interventions for all family members, **(10)** 2 day residential retreat, **(11)** Multidisciplinary professionals, **(12)** 5, **(13)** Family systems model, **(14)** | **(15)** To reduce risk factors and increase resilience factors for child and family functioning, **(16)** Coping skills and family functioning, **(17)** Current “strain” on work and leisure assessed with questions designed to assess the amount of strain on caregivers occupational and leisure functioning attributed to their children’s arthritis; Symptom Checklist-90-Revised (SCLBO-R) to measure psychological distress. | Caregiver psychological distress did not change but caregiver strain in both work and leisure showed a statistically significant improvement. |
|  | Hamall 2014 | **(1)** RCT protocol, **(2)** Stage 1 – any hospitalized child, Stage 2 onwards children attending cystic fibrosis, diabetes, rheumatology, gastroenterology clinics following **(3)** Australia, **(4)** Any primary caregivers, **(5)** Up to 18 years old, **(6)** Stage 1, Leaflet (Family Wellbeing and Resilience Factsheet), Stage 2, family wellbeing and resilience activity booklet, Stage 3, Parent Information Support Group for distressed parents who have been identified during step 2, which includes Face-to-Face and Online content, **(7)** Step 1 = 1050, Step 2 = Aiming to recruit 353 for a minimum sample size of 98 at 3 month follow-up, Step 3 = 120, **(8)** 6 months | **(9)** The Child Illness and Resilience Program (CHiRP), **(10)** Will vary depending on stage. In stage 3 participants will attend an initial 5 hours of face-to-face session, and a follow-up session (length not specified), as well as online forums (not specified) **(11)** Trained facilitators with a background in psychology or child health, **(12)** 5, **(13)**Family Resilience Theory (Walsh 2002) and behavior change (Padesky and Mooney 2012), **(14)** Family Resilience Theory formally integrates behavior / cognitions and systems approaches | **(15)** To improve the resilience and wellbeing of families caring for a child living with chronic illness, **(16)** Resilience and wellbeing, **(17)** The Kessler Psychological Distress Scale (K10); The McMasters Family Assessment Device (FAD); The Medical Outcomes Study Social Support Survey (MOSSSS); ‘maintaining a positive outlook’ and ‘ability to make meaning of adversity’ designed to measure Walsh's Family Resilience Framework (Walsh 2003; Sixbey 2008). | N/A as protocol |
|  | Hancock  2016 | **(1)** Pilot RCT , **(2)** Single ventricular defects, inpatients, **(3)** US, **(4)** Pregnant mothers of fetuses with a pre-natal diagnosis of single-ventricle heart disease planned to undergo palliative surgery during the neonatal period, **(5)** Neonates, **(6)** Consultation prior to surgery, **(7)** 40, **(8)** to hospital discharge | **(9)** Early palliative care, **(10)** Pediatric palliative care team, **(11)** One initial 45-90 minute consultation, followed by 30 minute follow-up appointments as needed. Total number of contacts ranged from 2 – 8 with a median of 3, **(12)** 3, **(13)** Staged palliation **(14)** Behavioral and cognitive approach, implied through description of counselling intervention | **(15)** To reduce maternal distress through early pediatric palliative care, **(16)** Maternal distress, **(17)** Beck Depression Inventory-II (BDI2); State Trait Anxiety Inventory (STAI); Brief Cope Inventory (BCI); PedsQL Family Impact Module (PedsQL) | Results were mixed. Early palliative care resulted in decreased maternal anxiety, improved maternal positive reframing, and improved communication and family relationships suggesting decreased overall maternal stress. However many sub-scales of the PedsQL showed poorer scores for intervention parents. However as a small pilot study this is underpowered and should be interpreted with caution. |
|  | Hernandez  1998 | **(1)** RCT, **(2)** Any condition attending clinics, **(3)** US, **(4)** Parents, **(5)** Not specified, **(6)** Group sessions, **(7)** 20, **(8)** to last session | **(9)** Self-applied relaxation techniques, **(10)** 8 hours, **(11)** Nurse, **(12)** 5, **(13)** King's System's Model of Nursing (1981), **(14)** Eclectic approach combining behavioral cognitive with systems approaches as there is explicit reference to King's System's Model of Nursing (a system theory) but aim of intervention is to for nurse to teach self-applied relaxation techniques (behavioral / cognitive) | **(15)** To reduce parental anxiety through parental education in self-relaxation techniques, **(16)** Coping, burnout, stress, anxiety (state and trait), **(17)** State Trait Anxiety Inventory (STAI); researcher developed demographic sheet, researcher developed relaxation experiences tool | No significant difference between groups after intervention. |
|  | Ireys  2001 | **(1)** RCT, **(2)** Any condition in community, **(3)** US, **(4)** Mothers, **(5)** 7-11 years, **(6)** Peer support through telephone contacts, face-to-face visits, and special family events, **(7)** 161, **(8)** 1 year | **(9)** Peer support, **(10)** Variable, **(11)** Experienced mothers of children with chronic conditions, **(12)** 7, **(13)** Social support – authors own description, **(14)** Systems approach based on authors own theoretical description of social support | **(15)** To enhance mothers’ perceived availability of social support, children’s self-esteem, and reduce parent’s psychological symptoms, **(16)** Anxiety, depression, stressful life events, **(17)** Maternal physical health (authors own question); Psychiatric Symptom Index (measures anxiety) (PSI); Beck Depression Inventory (BDI); Psychiatric Epidemiology Research Interview Life Events Scale (PERILES). | Experimental group reported lower levels of anxiety post-intervention compared with baseline scores. Stronger affect for mothers with higher anxiety levels pre-intervention. Participants in the control group reported higher levels of anxiety 1 year after baseline. The intervention had no demonstrable effect on symptoms of depression. |
|  | Ireys  1996 | **(1)** RCT, **(2)** Juvenile Rheumatoid Arthritis in community, **(3)** US, **(4)** Mothers, **(5)** 2-11 years, **(6)** Peer support, Telephone contacts, face-to-face visits, and special family events, **(7)** 53, **(8)** 15 months | **(9)** Peer support, **(10)** Variable, **(11)** Mothers of daughters age 18-24 who had had JRA since childhood, **(12)** 7, **(13)** Social support – authors own description, **(14)** Systems approach based on authors own theoretical description of social support | **(15)** To reduce maternal psychological symptoms with hypothesis that there would be particularly strong effects for mothers with elevated anxiety, depression or stressful life events, (**16)** Maternal mental health with focus on anxiety, depression and stressful life events, **(17)** Maternal physical health (authors own question); Psychiatric Symptom Index (measures anxiety) (PSI); Beck Depression Inventory (BDI); Psychiatric Epidemiology Research Interview Life Events Scale (PERILES). | Participants in the experimental group reported lower levels of anxiety post-intervention compared with baseline scores and mean anxiety scores decreased in each diagnostic group. Participants in the control group reported high levels of anxiety 1 year after baseline. The intervention was especially effective for mothers with high levels of anxiety at baseline. The intervention had no effect on depression, and no differential effects based on stressful life events, and no support for a dose-response relationship. |
|  | Jerram  2005 | **(1)** Wait-list controlled RCT, **(2)** Current serious health condition in community **(3)** New Zealand, **(4)** Parents, **(5)** Any, **(6)** Group sessions, **(7)** 58, **(8)** 6 months | **(9)** Support, skill development and stress management, **(10)** ≥ 6 hours, **(11)** Researcher with peer experience, **(12)** 6, **(13)** Social support, parental confidence working alongside professionals, child development framework, **(14)** Eclectic theoretical approach implied as focus of intervention is support, learning of skills and stress management (therefore spanning systems and behavior / cognitions) but no theories are referenced, or discussion of how these theoretical approaches have been integrated. | **(15)** To help parents manage illness related stress and learn advocacy and practical skills, **(16)** Social support, skills acquisition, stresses management, **(17)** Parent self-rating scale (PSRS); Affectometer 2 (A2) (wellbeing and happiness scale); Life Orientation Test (LOT) (general optimism); Family Environment Scale (10 dimensions of family life) (FES); Coping Health Inventory for Parents (COPE) (general coping); State Trait Anxiety Inventory -state scale only (STAI-state). Goal setting was also used as a measure with achievement of goals assessed | 17 / 38 variables showed statistically significant results, all in a ‘healthy’ direction. Non-significant results are not reported making interpretation difficult. The group process and session helpfulness received positive appraisals, personal goals were attained at high levels, and most participants said they would recommend the program to others in similar situations. Results should be interpreted with caution there are multiple statistical test, no pre-specified primary outcome and no sample size calculation reported. |
|  | Kaslow  2000 | **(1)** RCT, **(2)** Sickle cell disease, outpatients **(3)** US, **(4)** Parents, **(5)** 7-16, **(6)** Group sessions, **(7)** 59, **(8)** 6 months | **(9)** Manual based group family psychoeducation, **(10)** 6 hours, **(11)** psychologist and peers, **(12)** 6, **(13)** Stress-coping-adjustment framework (Lazarus and Folkman 1984) **(14)** Explicit behavioral / cognitive approach | **(15)** To enhance disease knowledge, and improve the psychological and psychosocial functioning of youth with SCD and their primary caregivers, **(16)** Psychological and psychosocial adjustment and disease knowledge, **(17)** Sickle Cell Disease Knowledge Test (SCDKT); Family Adaption and Cohesion Evaluation Scale (FACES 2); Dunst Family Support Scale (FSS); Dunst Family Resource Scale (FRS); investigator developed parental evaluation of intervention | The intervention yielded more improvements in child and primary caregiver disease knowledge than did the treatment as usual group, and the experimental group maintained this knowledge at the 6 month follow-up. No other statistically significant findings. |
|  | Kieckhefer  2014 | **(1)** RCT, **(2)** Any condition, outpatients, **(3)** US, **(4)** Parents, **(5)** 2-11 years, **(6)** Group sessions, **(7)** 129, **(8)** 6 months | **(9)** Building on Family Strengths Program, based on the chronic disease self-management course (Lorig, Sobel, Stewart, Brown, Bandura, Ritter, et al., 1999), , **(10)**7 X 2 hour weekly sessions, **(11)** Health professionals and peers, **(12)** 7, **(13)** Chronic Disease Self-Management theory based on self-efficacy enhancement, **(14)** Explicit cognitive / behavioral approach | **(15)** To improve parent self-efficacy in managing their child’s long term condition, **(16)** Parental self-efficacy, coping, parent-child shared management, perceived family quality of life, and depressive symptoms, **(17)** Investigator developed self-efficacy scale; Investigator developed shared management scale; Family Crisis Oriented Personal Evaluation Scale (F-COPES); Center for Epidemiologic Studies Depression Scale (CES-D 10); Quality of life (family impact scale) (QOL-FIS). | Intervention parents had higher self-efficacy scores, coping with child illness scores, parent-child shared management of the condition, family quality of life and lower scores for depressive symptoms at the 6 month end point. Average effect sizes were modest across outcomes. The greatest improvements came to parents in the least favorable quartile. |
|  | Leonard  2004 | **(1)** Evaluation via a web-based survey, **(2)** Rett Syndrome in community, **(3)** Australia, **(4)** Parents, relatives and other carers, **(5)** Any, **(6)** Email listserv, **(7)** 119, **(8)** Variable | **(9)** Online peer support, **(10)** Variable, **(11)** Peers via email, **(12)** 3, **(13)** No formal theory, isolation mentioned, **(14)** Unknown approach | **(15)** To reduce carer isolation and improve information sharing, **(16)** None, **(17)** Perceived advantages and disadvantages of the listserv, overall rating of usefulness, and reasons for satisfaction or dissatisfaction | 81.5% of participants felt Rettnet provided helpful advice concerning their child’s management. Rettnet was also useful in dealing with their child’s education and as a source of carer support. They rated it highly (mean 8.1 on a scale of 1 to 10), and the most common reason given for recommending the service to other parents was the emotional support provided. |
|  | Lewis  1991 | **(1)** RCT, **(2)** Epilepsy, outpatients, **(3)** Chile, **(4)** Parents, **(5)** 7-14 years, **(6)** Group sessions, **(7)** 252, **(8)** 5 months | **(9)**Family focused educational program incorporating decision-making and counselling components, **(10)** 6 hours in four sessions, **(11)** Not stated, **(12)** 4, **(13)** Roger's (1951) Helping and Counselling Model, **(14)** The intervention has a mix of behavioral / cognitive and systems elements integrated formally within Roger’s model. | **(15)** To support parent’s emotional adjustment to epilepsy, and improve epilepsy knowledge and decision making skills in order to facilitate parenting behaviors that support reduced restriction of activities and increased child participation in decision making, **(16)** Anxiety, **(17)** Parents’ perceptions of problems, sources of support and relationships with other children assessed using researcher developed tool; Taylor's Manifest Anxiety Scale (TMAS); Pencil and paper open ended questionnaire on impact of program. | Statistically significant reduction in anxiety levels in experimental group, largest effect size for mothers. Parenting style remained unchanged. |
|  | Li 2010 Mandarin | **(1)** RCT, **(2)** Lupus erythematosus and nephritis in hospital, **(3)** Beijing, China, **(4)** Parents, one per child, **(5)** Any, **(6)** One-to-one teaching, **(7)** 75, **(8)** 3 | **(9)** Education, **(10)** Not mentioned, **(11)** Doctor, **(12)** 5, **(13)** No theory mentioned, **(14)** | **(15)** To improve understanding of disease and self-management, **(16)** Knowledge and self-management, **(17)** Knowledge of disease and self-management – researcher developed tool; Qualitative feedback; Coping Health Inventory (CHIP); Anxiety (SAS); Depression (SDS) | Mixed – Coping improved in intervention parents but not anxiety and depression. Parents reported that they were not always comfortable with joint education approaches as they did not always want their children to know that was really happening. |
|  | Lind 2012 | **(1)** RCT abstract only, **(2)** Rare diseases, not specified, **(3)** Denmark, **(4)** Families, **(5)** Not specified, **(6)** Family residential course, one-day parent follow-up, closed internet forum for parents, **(7)** 16 families, **(8)** 6 months | **(9)** Family Empowerment Program, **(10)** > 15 hours, **(11)** Professionals, peers, **(12)** 4, **(13)** None stated, **(14)** Eclectic approach implied as a number of interventions components spanning systems and behavioral / cognitive approaches are listed. | **(15)** To empower families by offering an opportunity to create supportive networks, exchange of experience-based knowledge of social and legal affairs, knowledge of psychological aspects of family life and tools for conflict management and goal setting, **(16)** Empowerment, knowledge of child's disease, the social welfare system and legal aspects, benefits from networking and experience based knowledge sharing, **(17)** Not specified (abstract only) | Preliminary results show that participators increased their knowledge in different areas, benefitted from creating network with each other and benefitted from sharing experience-based knowledge. The qualitative evaluation showed that families during experienced cohesion and unity. The physical meeting enabled them to learn from each other and from the instructors’ professional knowledge. Participation in RFD created drive and hope for the future. Interpret with caution as abstract only and data behind results not clear. |
|  | Lindstrom  2016 | **(1)** 1 arm evaluation , **(2)** Type 1 diabetes and irritable bowel disease in outpatients, **(3)** Sweden, **(4)** Parents, **(5)** 1-18, **(6)** Group sessions, **(7)** 16, **(8)** 6 months | **(9)** CBT, **(10)** ≥ 8 hours, **(11)** Counsellor, **(12)** 5, **(13)** Based on CBT methods and systemic theory, **(14)** Integrated behavioral / cognitive and systems approach | **(15)** To reduce parental clinical burnout by focusing on coping with long term stress and explore the acceptability of the intervention, **(16)** Clinical burnout, performance based self-esteem (PBSE), **(17)** Shirom–Melamed burnout questionnaire (SMBQ); Performance-based self-esteem scale (PBSE). | Based on very small numbers parents’ subjective evaluations were mainly positive, and SMBQ and PBSE scale measurements were significantly reduced, with effects remaining 6 months after completion of the intervention. |
|  | Lohan  2016 | **(1)** RCT protocol, **(2)** Type 1 diabetes in community, **(3)** Australia, **(4)** Parents, **(5)** 2-10 years, **(6)** Group sessions, **(7)** 60, **(8)** 6 months | **(9)**Positive parenting for healthy living (Triple P) , **(10)** 4 hours, **(11)** Trained Triple-P practitioner, **(12)** 6, **(13)** Triple-P parenting - based on social learning principles, cognitive behavioral and developmental theory, **(14)** Explicit behavioral / cognitive approach | **(15)** To improve positive parenting practices in parents of children with Type 1 diabetes, **(16)** Parenting skills and confidence with both general parenting and illness management, parental self-regulation, positive parenting practices, parent self-care, **(17)** Self-Efficacy for Diabetes Scale (parenting efficacy) (SED); Alabama Parenting Questionnaire (Parenting behavior) (APQ); Parent Experience of Child Illness Scale (PECIS); Parenting Stress Index-Short Form (PSI-SF); Child & parent behavior (home observation). | N/A as protocol |
|  | Marteau 1987 | **(1)** 1 arm evaluation, **(2)** Diabetes, **(3)** UK, **(4)** Parents, **(5)** 3-21 years, **(6)** Group sessions, **(7)** 97, **(8)** 3 months | **(9)** Residential weekend, **(10)** ≥ 10 hours, **(11)** Psychologists, physicians, a social worker and a dietician, **(12)** 5, **(13)** None mentioned, **(14)** Unknown | **(15)** To help parents to learn and share approaches to identifying common problems and put these ideas in to practice, **(16)** Problem solving, **(17)** Perceived difficulty on managing child's condition (researcher developed questionnaire); confidence looking after child (researcher developed questionnaire). | Parents rated themselves significantly more confident in looking after their child immediately following the weekend, although ratings of perceived difficulty in managing their child's condition did not alter. Three months later parents' confidence had further increased, although perceived difficulty was unchanged. |
|  | Mattsson 1972 | **(1)** Qualitative evaluation, **(2)** Hemophilia, **(3)** US, **(4)** Parents, **(5)** 3-19 years, **(6)** Discussion groups, **(7)** 10, **(8)** 2 years | **(9)** 25 weekly Group meetings, **(10)** 37.5 hours, , **(11)** a general and child psychiatrist **(12)** 6, **(13)** None explicitly stated, **(14)** Implied cognitive and behavioral approach | **(15)** Not clearly stated, but suggests improvement of parent-child relationship. **(16)** Not clearly stated, but suggests improving parent understanding and emotional distress**(17)** Qualitatively evaluated | The author state parents showed a continued improvement in self-confidence as parents of chronically ill children. |
|  | Melnyk  1994 | **(1)** RCT, **(2)** Hospitalized children, **(3)** US, **(4)** Mothers, **(5)** 2-6 years, **(6)** Recorded information given on ward, **(7)** 127, **(8)** 2 weeks | **(9)** Parent education, **(10)** 14 minutes, **(11)** Recorded information, **(12)** 6, **(13)** Coping; self-regulation theory; Control theory, **(14)** Behavioral / cognitive approach, explicit | **(15)** To improve maternal coping with an unplanned hospital admission in a young child, **(16)** Maternal confidence; anxiety and participation in child’s care, **(17)** State Trait Anxiety Inventory (STAI); Index of Parent Participation / Hospitalized Child (Parent participation in care) (IPP); Index of Parental Support during Intensive Procedures (IPS); Parental Belief Scale (PBS). | Mothers in all intervention groups reported significantly less anxiety participated in more care and gave more support during intrusive procedures and reported less maternal anxiety post-hospitalization than controls, with mothers who received both types of information reporting the least anxiety and offering more support. |
|  | Melnyk  1997 | **(1)** Pilot RCT , **(2)** Children in PICU, **(3)** US, **(4)** Mothers, **(5)** 1-6 years, **(6)** Taped and written information, parent-child activity book, **(7)** 30, **(8)** 4 weeks post discharge | **(9)** Education / behavioral (COPE), **(10)** Varies, **(11)** Recorded and printed information, **(12)** 6, **(13)** Self-regulation theory; control theory; the emotional contagion hypothesis, **(14)** Behavioral / cognitive approach, explicit | **(15)** To improve coping in critically ill children and their mothers, **(16)** Parent understanding of their hospitalized children and their role; maternal anxiety, negative mood and PTSD symptoms; mothers’ participation in care, **(17)** Index of Parent Support during Intrusive Procedures (IPS); Index of Parent Participation/Hospitalized Child (IPP); State–Trait Anxiety Inventory (STAI); Profile of Mood States (POMS); Mothers’ stress related to the PICU environment was measured by the Pediatric Stressor Scale: Pediatric Intensive Care (PSS:PICU); Mothers’ posttraumatic stress symptoms were measured with the Post-Hospital Stress Index for Parents (PSI-P), developed for this study; The Parenting Role Questionnaire (PRQ). | Based on a small sample, mothers who received the COPE program: (a) provided more support to their children during intrusive procedures; (b) provided more emotional support to their children; (c) reported less negative mood state and less parental stress related to their children’s emotions and behaviors; and (d) reported fewer post-traumatic stress symptoms and less parental role change four weeks following hospitalization. |
|  | Melnyk  2004 | **(1)** RCT, **(2)** Children admitted to PICU with no prior admissions who were expected to survive, **(3)** US, **(4)** Mothers, **(5)** 2-7 years, **(6)** Taped and written information, parent-child activity book, telephone call post discharge, **(7)** 174, **(8)** Follow-up assessments at 1, 3, 6 and 12 months following discharge | **(9)** Education / behavioral (COPE), **(10)** Unclear, **(11)** Recorded and printed information, **(12)** 5, **(13)** Self-regulation theory; control theory; the emotional contagion hypothesis, **(14)** Behavioral / cognitive approach, explicit | **(15)** To strengthen mother's beliefs and knowledge of typical behaviors and emotions of young children as they recovered from critical illness, and to enhance their ability to facilitate their children's adjustment, **(16)** Knowledge and understanding of typical child behaviors and emotions during and after hospitalization; parent participation in emotional and physical care, **(17)** State Anxiety Inventory (A-State); Profile of Mood States (POMS); Depression (POMS dep); Pediatric Stressor Scale: Pediatric Intensive Care (PSS:PICU); Post Hospitalization Stress Index for Parents (PSI-P); Involvement in Physical Care (VAS-PC); Involvement in Emotional Care (VAS-EC); Index of Parent Participation / Hospitalized Child (IPP); Parental Belief Scale (PBS). | COPE mothers were significantly more involved in physical and emotional care, less stressed regarding their children’s overall appearance, and reported less total negative mood 1 year after hospitalization. They also reported less total stress after transfer to the general pediatric unit, and regarding medical procedures, their children’s behaviors and emotions, less negative mood and depression 1 month after hospitalization, and fewer PTSD symptoms 6 months after hospitalization compared to controls. |
|  | Minor  2006 | **(1)** Observational before / after study, **(2)** Any condition attending medical clinics, **(3)** Canada, **(4)** Caregivers **(5)** 3-18 years, **(6)** Group sessions, **(7)** 44, **(8)** to last session, 8 weeks | **(9)** Mindfulness based stress reduction and yoga, **(10)** 16 hours, **(11)** Clinical social worker and family physician, **(12)** 7, **(13)** Mindfulness-based stress reduction, **(14)** Explicitly behavioral / cognitive | **(15)** To provide tools for self-care, **(16)** Stress and mood disturbance, **(17)** Symptoms of stress inventory (SOSI); Profile of Mood States (POMS). | Prior to the intervention, caregivers reported very high levels of stress and mood disturbance. These decreased substantially over the 8-week program, with an overall reduction in stress symptoms of 32% and total mood disturbance of 56%, with significant results in a positive direction for all sub-scales. Description of group sizes suggests there may have been a significant drop-out rate. |
|  | Morawska  2017 | **(1)** RCT, **(2)** Asthma and eczema in community, **(3)** Australia, **(4)** Parents, **(5)** 2-10 years, **(6)** Group sessions, **(7)** 107, **(8)** 6 months | **(9)** Positive Parenting for Healthy Living, based on the Triple P Positive Parenting Program, **(10)** 4 hours, **(11)** Trained Triple-P Practitioner, **(12)** 8, **(13)** Triple-P Parenting, based on social learning principles, cognitive behavioral and developmental theory, **(14)** Explicitly based on a behavioral / cognitive approach | **(15)** To improve positive parenting practices in parents of children with asthma and / or eczema, **(16)** Parenting skill and confidence relating to both general child behavior, and specific to illness management, parenting practices, parenting and family stress, **(17)** Parenting Scale (PS); Eyberg Child Behavior Inventory (ECBI); Child Adjustment and Parent Efficacy Scale - Emotional Maladjustment Scale only (CAPES). | Overall parent-reported ineffective parenting as well as parental over reactivity reduced as a result of intervention. Parent report of child behavior problems also decreased, but there were no changes in children’s emotional adjustment. No changes in observed parent or child behavior were found. Stress reduced for parents in the intervention group compared to the CAU group, but there were no changes in parental anxiety or depression. Effects showed evidence of reliable and clinical changes and were maintained at 6-month follow-up. |
|  | Nicholas  2007 | **(1)** Mixed Methods Evaluation, **(2)** Technology assisted children with lung disease in community, **(3)** Canada, **(4)** Parents and family caregivers, **(5)** 2 months to 6.5 years, **(6)** One-to-one peer matching, **(7)** 34, **(8)** 4 months | **(9)** Matched peer support delivered in person, by phone and via the internet, **(10)** Encouraged to remain in contact once per week, **(11)** Peers, **(12)** 6, **(13)** Social support, **(14)** Systems approach explicitly stated in a nuanced description of social support and intended benefits | **(15)** To match parents with similar caregiving responsibilities to reciprocally engage in parent-to-parent support, **(16)** Coping, social isolation, illness meaning **(17)** Pre- and post-intervention questionnaires followed by qualitative interviews with a subset of parents. Content not well described but these included the Meaning of Illness Questionnaire (MIQ) to measure clinical changes in participant coping, social isolation and illness intrusion (meaning of illness). | No statistically significant changes in parent coping, meaning of illness, or social isolation. Qualitative analysis indicated benefits including mutual sharing reduced isolation, increased knowledge, and feeling understood. Challenges included scheduling difficulties and personality incompatibility. |
|  | Othman  2010 | **(1)** Non-randomized controlled trial , **(2)** Cancer, hospital and community based, **(3)** Malaysia, **(4)** Parents, **(5)** Any, **(6)** Group sessions, **(7)** 79, **(8)** 4-8 weeks | **(9)** Psychoeducational program (PeP), **(10)** 450 min group psycho-educational sessions with 10–15 min review time after two sessions, **(11)** A doctoral student in clinical psychology and a pediatric oncologist, **(12)** 2, **(13)** Based on 4 theoretical concepts: self-regulation; control; planned behavior; emotional contagion theory, **(14)** Behavioral and cognitive approach with explicitly referenced theories | **(15)** To improve parents’ knowledge of cancer, increase psychological awareness and acceptance, recommend effective coping strategies, and decrease anxiety and stress, **(16)** Parents’ knowledge of childhood cancer; parents’ psychological awareness and acceptance; coping; anxiety; stress, **(17)** The Knowledge Acquisition (KA) questionnaire; Perceived Acquired Knowledge (KA-p); State Anxiety subscale of a reliable widely used instrument, The State-Trait Anxiety Inventory (STAI)(SA); Strain Questionnaires (SQ); Parents’ Activities with Children (PA) | There was increased knowledge about cancer in the intervention parents compared with standard care. Intervention parents reported reduced anxiety and increased activities with children after the program; however, differences were not significant. This trial was not randomized so results should be interpreted with caution. |
|  | Palermo  2016 | **(1)** Pilot RCT , **(2)** Chronic pain, present for at least 3 months, interfering with daily functioning, clinic based, **(3)** US, **(4)** Parents, **(5)** 11-17 years, **(6)** Individual sessions and parent manual, **(7)** 61, **(8)** 3 months | **(9)** Problem solving skills training (PSST), **(10)** 4-6 hours plus time to complete a worksheet and take home weekly assignments, **(11)** Postdoc psychology fellows trained in chronic pain management, **(12)** 8, **(13)** Problem Solving Skills Training (PSST) based on the social problem solving model, (D'Zurilla and Nezu 1999 and 2007), **(14)** | **(15)** To improve parental mental health symptoms, health and well-being, and adaptive parenting, **(16)** Mental health symptoms, health and wellbeing, adaptive parenting, **(17)** Beck Depression Inventory-II (BDI-II); The Profile of Mood States-Standard (POMS); Bath Adolescent Pain–Parental Impact Questionnaire (BAPQ-PIQ); Pain Catastrophizing Scale for Parents (PCS-P); Short Form Health Survey 12 (SF-12); The Parenting Stress Index-Short Form (PSI-SF); Helping for Health Inventory (HHI); Social Problem-Solving Skills Inventory– Revised (SPSI-R) | Problem Solving Skills Training (PSST) was associated with reduced parental depression, improved general mental health, and reduced pain catastrophizing. However this is a pilot study with a sample size powered to detect a large change in depressive symptoms only and the results should be interpreted with caution. |
|  | Rayner  2016 | **(1)** RCT protocol , **(2)** Life threatening conditions, 4-10 months after diagnosis **(3)** Australia, **(4)** Caregivers, **(5)** 0-18, **(6)** Group sessions via videoconferencing, **(7)** 184, **(8)** 6 months | **(9)** Acceptance Commitment Therapy (ACT), **(10)** 7.5 hours, **(11)** Psychologists and mental health clinicians trained in ACT with a trauma focus, **(12)** 7, **(13)** Take a Breath logic model based on Acceptance and Commitment Therapy, **(14)** Explicit behavioral / cognitive approach | **(15)** To reduce parental distress, including posttraumatic stress symptoms (primary outcomes) in parents of children with life-threatening illness or injury, **(16)** Distress, psychological skills, adjustment, **(17)** Acceptance and Action Questionnaire – II (AAQ-II); Parental Psychological Flexibility Questionnaire (PPFQ); Five Facet Mindfulness Questionnaire – Short Form (FFMQ-SF); Valuing questionnaire (VQ); Posttraumatic Stress Disorder Checklist – Specific (PCL-S); Depression Anxiety Stress Scale (DASS); World Health Organization Quality of Life – BREF (WHOQol – BR); Posttraumatic Growth Inventory – Short Form (PTGI-SF); Parent Experience of Child Illness Scale (PECI); Family Management Measure (FaMM). | N/A as protocol |
|  | Ridge 2014 | **(1)** Mixed methods feasibility study, **(2)** Type 1 diabetes for at least the past year, **(3)** UK, **(4)** Carers, **(5)** 10-18, **(6)** Group sessions, **(7)** 31 parents of young people, **(8)** 13 weeks | **(9)** Diabetes-Oriented Learning Family Intervention (DOLFIN), **(10)** 9 hours, **(11)** Not clear, **(12)** 7, **(13)** Motivational interviewing; trans-theoretical model of change, **(14)** Behavioral and cognitive approach, implied by reference to the trans-theoretical model, though whether it was used inform intervention development is not stated | **(15)** To involve parents in diabetes care, empower carers as collaborators in diabetes management, and to increase positive interactions between parent and child, **(16)** Positive interactions between parent and child, **(17)** Patient Health Questionnaire 9 (measures depression) (PHQ-9); Generalized Anxiety Disorder 7 (GAD7); Diabetes Family Conflict Scale (DFCS); Diabetes Family Responsibility Questionnaire (DFRQ) | Carers felt the most helpful aspect of DOLFIN was the opportunity to meet other parents in similar situations. There were non-significant improvements in depression, family conflict, and child autonomy, and a non-significant deterioration in anxiety. This is a pilot study not powered to detect a significant change and results should be interpreted with caution. |
|  | Ritchie  2000 | **(1)** Qualitative evaluation, **(2)** Cystic fibrosis, spina bifida or diabetes, community based, **(3)** US, **(4)** Parents, **(5)** 0-7 years, **(6)** Peer support via group telephone sessions **(7)** 137, **(8)** 6 months | **(9)** Peer support group via telephone, **(10)** 12 hours, **(11)** Peers (mothers of older children with the same condition) and professionals, **(12)** 7, **(13)** Social support, adaptation and coping, **(14)** Explicit systems approach | **(15)** To improve parents’ protective factors related to stress, support, and coping; outcomes of parental health and competence; and child resilience, **(16)** Stress, coping, health and competence, **(17)** Qualitative evaluation | Parents described increased social support, enhanced coping and sense of confidence, and changed outlook, family activities and relationships, and health care encounters. The vast majority were satisfied with the support groups. Generally, the parents seemed to describe the intervention’s impact most powerfully in their weekly diaries or in the telephone interviews immediately following the completion of the support groups. Many parents attributed the positive effects of the groups, such as increased confidence, changed perspectives, decreased isolation, and changed family activities and health care encounters, to the opportunity to share and compare feelings and ways of doing things. |
|  | Sadeghi Shabestari 2013 | **(1)** RCT abstract only , **(2)** Asthma, **(3)** Iran, **(4)** Mothers, **(5)** 6-14 years, **(6)** Instruction (not specified) and ongoing mother to child massage, **(7)** 60, **(8)** Not mentioned | **(9)** Mother to child massage training, **(10)** 15 hours, **(11)** Not stated, **(12)** 3, **(13)** None stated, **(14)** Unknown, not enough information available | **(15)** To reduce anxiety levels in mothers of asthmatic children, **(16)** Anxiety, **(17)** State-trait Anxiety Inventory (STAI) | Maternal anxiety levels decreased following combined massage therapy. |
|  | Sahler 2002 | **(1)** RCT, **(2)** Cancer, **(3)** US, **(4)** Mothers, **(5)** Not specified, **(6)** One-to-one sessions, **(7)** 92, **(8)** 3 months | **(9)** Problem-solving Skills Training, **(10)** 8 hours, **(11)** A mental health professional with a master's degree, or a doctoral candidate in psychology, **(12)** 7, **(13)** Problem Solving Skills Training (PSST) based on the social problem solving model (D'Zurilla and Nezu 1999 and 2007), **(14**) Behavioral / cognitive approach explicitly based on the social problem solving model. | **(15)** To reduce stress levels and increase levels of self-satisfaction and wellbeing, (**16)** Stress, self-satisfaction, wellbeing, **(17)** The Social Problem-Solving Inventory-Cancer (SPSI-C); Profile of Mood States (7 subscales: tension / anxiety, Depression / Dejection, Anger / Hostility, Fatigue, Confusion, Vigour, Friendliness) (POMS). | Mothers in the PSST group had significantly enhanced problem solving skills ad significantly reduced negative affectivity compared with controls. PSST had the greatest impact on improving constructive problem solving, whereas improvement in mood was most influenced by decreases in dysfunctional problem solving. |
|  | Schwartz 2004 | **(1)** RCT, **(2)** Hematology and oncology inpatients (mostly cancer and sickle cell disease), **(3)** US, **(4)** Primary caregivers, **(5)** Any, **(6)** Individual writing exercise, **(7)** 72, **(8)** 4 months | **(9)** Written emotional disclosure about the most stressful experiences they have had, **(10)** 1 hour, **(11)** N/A, **(12)** 5, **(13)** Written emotional disclosure (In Schwartz 2004 linguistic paper), **(14)** Behavioral and cognitive approach, explicitly referenced | **(15)** To improve affect regulation, including regulation of emotion-related experience and physiological responses, to improve psychological and physiological function, **(16)** Distress, **(17)** Mood and Anxiety Symptom Questionnaire (MASQ);Profile of Mood States Short Form (POMS); 36-Item Short Form Survey (SF-36); Caregiver Appraisal Scale (CAS); Penneker's Physical Symptom Scale (PPSS); Brief Mood Rating Scale (BMRS). | The experimental group experienced less positive affect, more negative affect, and physical symptoms immediately following writing than the control group, as hypothesized. However, in the long term the experimental group also had less vitality than the control group suggesting that this intervention is not effective for improving long term wellbeing. |
|  | Staab 2010  German | **(1)** RCT, **(2)** Eczema, **(3)** Germany, **(4)** Parents, **(5)** Any, **(6)** Group sessions, **(7)** Not stated, **(8)** 1 year | **(9)** Eczema education, **(10)** Not clear, **(11)** Multidisciplinary team, **(12)** 5, **(13)** None stated, **(14)** | **(15)**, To improve medical outcomes, reduce parental and child illness burden, improving self-efficacy (parent and child), **(16)** Parental self-efficacy, coping, **(17)** Acceptance of illness, emotional coping, impact on social life, psychosomatic wellbeing, disease knowledge (measurement tools not described - it is not clear whether these outcomes relate to parents or only children | Interpret with caution. Authors state that the intervention was very effective but reporting is not detailed. |
|  | Stabler 1981 | **(1)** RCT abstract only , **(2)** Cystic fibrosis, **(3)** US, **(4)** Parents, **(5)** Any, **(6)** Group sessions, **(7)** 13 families, **(8)** 6 months | **(9)** Communication skills training, **(10)** > 4 hours, **(11)** Not stated (abstract only), **(12)** 2, (**13)** None stated, **(14)** Unknown approach, not mentioned. | **(15)** Not stated, **(16)** Not stated, **(17)** Family interaction, child adjustment, locus of control, and understanding of CF (tools not specified) | No differences between groups from the first to the final data collection times; communication training did not improve family functioning. Abstract only available so limited information. |
|  | Starks 2016 | **(1)** Cluster RCT , **(2)** PICU admission over 8 days, **(3)** US, **(4)** Parents, **(5)** Any, **(6)** Group sessions, **(7)** 380 family members, **(8)** 3 months | **(9)** Communication training with structured ethics consultation template, based on palliative care principles **(10)** Varies, **(11)** Pediatric Advanced Care Team members (who included 2 nurses, 2 social workers, 3 chaplains, and a child life specialist), **(12)** 7, **(13)** Patterson's theory of Family Adjustment and Adaptation Response (FAAR) adapted to incorporate elements of successful communication interventions, **(14)** Explicitly based on a systems theory approach. | **(15)** To reduce family members acute and long term stress symptoms, **(16)** Stress symptoms among family members, and clinician-family communication and decision making, **(17)** Family Relationships Index (FRI); the Multidimensional Scale of Perceived Social Support (MSPSS); Wake Forest Trust Scale to assess family perceptions of the physicians and nurses caring for their child (WFTS); the Patient Health Questionnaire 9 (PHQ-9) General Anxiety Disorder 7 (GAD7), Acute Stress Disorder Scale (ASDS), and the PTSD Checklist (PCLC) to assess symptoms of depression, anxiety, and acute- and post-traumatic stress disorder; Quality of Communication scale (QOC). | Psychosocial outcomes not reported yet. |
|  | Stewart 2011 | **(1)** Observational study , **(2)** Children with asthma and allergies from rural settings participating in an online support program, **(3)** Canada, **(4)** Parents, **(5)** 7-11 years, **(6)** Online, **(7)** 19, **(8)** Unclear | **(9)** Online peer support groups, **(10)** < 2 hours, **(11)** Counselling psychologist and peer mentor, **(12)** 4, **(13)** Not stated, **(14)** Implicitly based on a systems theory approach as social support discussed at length but no specific theory referenced. | **(15)** Not stated, **(16)** Not clear, **(14)** Qualitatively evaluated | Parents received information and reassurance from other parents in peer support sessions. Parents appreciated the accessibility and anonymity of the online support group. |
|  | Stuttard 2014 | **(1)** Non-randomized waiting list controlled trial, **(2)** Autism Spectrum Condition and/ or moderate/severe intellectual and/or complex disabilities, **(3)** UK, **(4)** Parents, **(5)** 3-12 years, **(6)** Group sessions, **(7)** 76, **(8)** Immediate, with a 6 month follow up for all who had received the intervention | **(9)** Parenting skills training, **(10)** 20 hours, **(11)** Two facilitators, one a clinical psychologist, **(12)** 8, **(13)** Behavioral theory and positive behavior management. Principles of experiential learning (Kolb, 1984) inform the training approach, **(14)** Explicit behavioral / cognitive approach | **(15)** To enable parents/carers to understand, and have the skills and confidence to manage, their child’s behavior in the context of additional needs, **(16)** Child behavior management, **(17)** Eyberg Child Behavior Inventory (ECBI); Parenting Sense of Competence Scale (PSOC). | Receipt of the intervention was associated with significant reductions in parent-reported behavior problems and significant improvements in parenting efficacy and satisfaction. At six month follow-up, progress towards achieving parent-set child behavior goals and parenting satisfaction had been maintained. |
|  | Svavarsdottir  2014 | **(1)** RCT secondary evaluation of only intervention arms of 3 trials, **(2)** Cancer, asthma and diabetes, in patients and outpatients **(3)** Iceland, **(4)** Families, **(5)** Any, **(6)** One-to-one face-to-face therapeutic conversation, **(7)** 60 parents from 37 families, 35 mothers and 25 fathers (8) 1 week after 2^nd^ interview | **(9)** Family genogram and ecomap, therapeutic questions, recommendations, **(10)** 15-50 minutes, **(11)** Nurses with family systems expertise, **(12)** 7, **(13)** Family Systems Nursing (FSN), Calgary Family Assessment (CFAM), Calgary Family Intervention (CFIM), **(14)** Integrated behavioral /cognitive and systems approach | **(15)** To improve perceived family support, expressive family functioning and diseases related QOL, **(16)** Family functioning, well-being, and quality of life (QOL), **(17)** Iceland Expressive Family Functioning Questionnaire (ICE-EFFQ); Iceland Family Perceived Support Questionnaire (ICE-PFSQ); Disease-Specific QOL Questionnaire | Mothers of the children / teenagers perceived significantly higher family support after the interventions compared with before; and also reported significantly higher collaboration and problem-solving abilities on the expressive family functioning scale. However, no significant differences were found on the fathers’ perceived family support or expressive family functioning following the interventions. |
|  | Svavarsdottir  2012 | **(1)** RCT, **(2)** Hospitalized children and outpatients, **(3)** Iceland, **(4)** Families, **(5)** Any, **(6)** One-to-one face-to-face therapeutic conversation, **(7)** 76, **(8)** 3-5 days post discharge | **(9)** Family therapeutic conversation, **(10)** 1 hour, **(11)** Study nurse, **(12)** 7, **(13)** Family Systems Nursing (FSN), Calgary Family Assessment (CFAM), Calgary Family Intervention (CFIM), **(14)** Integrated behavioral /cognitive and systems approach | **(15)** To improve reported family support and expressive family functioning, **(16)** Family functioning during healthcare episode, **(17)** Iceland Expressive Family Functioning Questionnaire (ICE-EFFQ); Iceland Family Perceived Support Questionnaire (ICE-PFSQ) | Mixed - Overall parents in the intervention group reported significantly higher perceived cognitive support but not emotional support compared to control parents. However sub-group analyses revealed this only applied to parents of acutely unwell children. Parents of chronically ill children reported significantly lower perception of family support post intervention compared to pre intervention. |
|  | Swallow 2014 | **(1)** Pilot RCT, **(2)** Chronic kidney disease, in community or hospital **(3)** UK, **(4)** Parents, **(5)** 0-19, **(6)** Online, **(7)** 55, **(8)** 20 weeks | **(9)** Interactive health communication, **(10)** Variable, **(11)** Peers, **(12)** 6, **(13)** Self-efficacy (Bandura), **(14)** Explicitly based on a behavioral / cognitive approach | **(15)** To assess feasibility of a full-scale RCT of OPIS and to investigate trends in outcome measures, **(16)** Home based care of children with LTCs. Expectation on parents to perform complex clinical care at home, **(17)** Family Management Measure (FaMM); Service System Subscale of Family Empowerment Scale (FES-SSS); Dads Active Disease Support Scale (DADS). | Intervention group parents showed a greater improvement in perceived competence to manage their child’s condition compared to control group parents. A full-scale RCT is feasible. |
|  | Tew  1997 | **(1)** RCT , **(2)** Long term conditions, outpatients, **(3)** US, **(4)** Parents, **(5)** 3-10 years, **(6)** Group sessions, **(7)**, 28, **(8)** To end of course | **(9)** Filial therapy, **(10)** 20 hours, **(11)** Counsellor and Child Life Specialists, **(12)** 6, **(13)** Filial therapy, an intervention aimed at strengthening and enhancing the parent-child relationship through child centered play therapy skills, **(14)** Behavioral / cognitive approach | **(15)** To increase parental acceptance of chronically ill young children, reduce stress levels, and decrease children’s emotional and behavioral problems, **(16)** Acceptance, stress, **(17)** The Parenting Stress Index (PSI); The Porter Parental Acceptance Scale (PPAS); The Child Behavior Checklist to measure perceptions of child behavior. | Intervention group parents significantly reduced their stress levels and increased their attitude of acceptance towards their children. |
|  | Tsuruta  2005 | **(1)** Mixed Methods Evaluation, **(2)** Hospitalized children, **(3)** Japan, **(4)** Family members caring for children in hospital, **(5)** Any, **(6)** Group exercise sessions in hospital during child’s admission, **(7)** 156 family members and 146 children, **(8)** to end of participation | **(9)** Structured exercise sessions, **(10)** Variable, **(11)** Not described, **(12)** 5, **(13)** The program is based on the principle that the healthy condition of family members is a pre-requisite in maintaining the patient’s physical and mental stability and is also the basis for family resilience, **(14)** Behavioral / cognitive approach implied as intervention seeks to change exercise behavior to ease physical tension, release stress and provide a diversion, thereby modifying cognitive processes through behavior | **(15)** To ease physical tension, release stress and provide a diversion, **(16)** Blood pressure and pulse rate, **(17)** Blood pressure and pulse; qualitative feedback | Qualitative findings suggest that participants valued the intervention; there was a small (non-clinically significant) reduction in average blood pressure between the start and end of the program. |
|  | Vera 2016 | **(1)** Observational study, **(2)** Children at point of asthma diagnosis, **(3)** Mexico, **(4)** Caregivers, **(5)** Not specified, **(6)** Training in use of adherence card to track asthma management behaviors, a single education / cognitive behavioral therapy session, and reinforcement of knowledge and techniques at follow-ups, **(7)** 50 recruited with 31 completing intervention, **(8)** 6 months | **(9)** Cognitive-behavioral intervention based on educational slides about asthma management and review of parent’s asthma management and behaviors, **(10)** 1.5. hours for face-to-face therapeutic session, with preparation and follow up contact, **(11)** Not stated, **(12)** 7, **(13)** Cognitive Behavioral Therapy, **(14)** Behavioral and cognitive approach referencing CBT | **(15)** To decrease anxious and depressive symptomatology in caregivers of children suffering allergic asthma, **(16)** Anxiety and depression, **(17)** Beck Depression Inventory II (BDI-II); Beck Anxiety Inventory (BAI) (both standardized for Mexican population); Card of adherence behaviors, author developed tool to enable parents to record their asthma management behaviors. | Results showed statistically and clinically significant improvements in depressive (mild to minimal) and anxiety symptoms (mild to mild-minimal). The size and effectiveness of the clinical effects were greater for depressive symptoms, and medium for anxious symptoms. Improvement occurred in all 31 participant caregivers and remained after six months of treatment completion. |
|  | Wacharasin  2015 | **(1)** Qualitative Evaluation, **(2)** Thalassemia, outpatients, **(3)** Thailand, **(4)** Parents and grandmothers, **(5)** 1-14 years, **(6)** Group sessions, **(7)** 25, **(8)** To end of intervention | **(9)** Family Empowerment Program, **(10)** To empower families, **(11)** Nurses skilled in advanced family nursing practice, **(12)** 6, **(13)** Illness Belief Model, **(14)** Behavioral and cognitive approach, explicitly referenced in terms of Illness Belief Model | **(15)** To empower families, **(16)** Family empowerment, **(17)** Qualitatively evaluated | Family caregivers reported that the FEP helped them share beliefs and experiences related to caring for their child with thalassemia, make decisions related to families’ problems/needs and beliefs, provide each other mutual social support, and develop increased ability to manage care for their chronically ill child through sharing information and learning from other family caregivers about family functioning, family management, and family relationships. |
|  | Wallace  2016 | **(1)** Pilot 1 arm evaluation, **(2)** Chronic Pain, outpatients, **(3)** US, **(4)** Mothers, **(5)** 13-18 years, **(6)** Group sessions, **(7)** 8, **(8)** 6 months | **(9)** Acceptance and Commitment Therapy, **(10)** 8 weekly sessions 75 minutes in length (10 hours total), **(11)** Not mentioned, **(12)** 5, **(13)** Psychological flexibility, a core construct of third-wave behavior therapies (also called “contextual cognitive behavioral therapies”), **(14)** Behavioral and cognitive approach, implied through reference to psychological flexibility | **(15)** To improve pain-related parent psychological flexibility, and reduce parent’s protective pain monitoring responses, **(16)** Psychological flexibility and parent’s protective responses to child pain, **(17)** Parent Psychological Flexibility Questionnaire (PPFQ); Adult Responses to Children’s Symptoms (ARCS); PROMIS pain interference; Treatment satisfaction | Parent pain-related psychological flexibility increased during the intervention and through follow-up. Protective parenting responses decreased significantly during follow-up only, as did adolescent-rated pain interference. Parents indicated high satisfaction with the intervention. |
|  | Worley  1991 | **(1)** RCT, **(2)** Spina Bifida, outpatients **(3)** US, **(4)** Parents, **(5)** Any, **(6)** Manual based counselling session, **(7)** 115, **(8)** up to 1 year | **(9)** Financial counselling, **(10)** 2-4 hours, **(11)** Financial counsellor, **(12)** 7, **(13)** None mentioned, **(14)** Cognitive / behavioral approach implied as intervention seeks to change financial management behavior | **(15)** To teach financial management skills, **(16)** Financial management measured by 68 variables (not described), **(17)** Researcher developed financial management and knowledge questionnaire | Improvements in 18/64 variables. ¾ families believed that the intervention had or would have a beneficial effect on their lives. |
